# Phosphate Derivatives of 3-Carboxyacylbetulin: SynThesis, In Vitro Anti-HIV and Molecular Docking Study

**DOI:** 10.3390/biom10081148

**Published:** 2020-08-05

**Authors:** Krzysztof Marciniec, Elwira Chrobak, Aleksandra Dąbrowska, Ewa Bębenek, Monika Kadela-Tomanek, Paweł Pęcak, Stanisław Boryczka

**Affiliations:** 1 Department of Organic Chemistry, Faculty of Pharmaceutical Sciences in Sosnowiec, Medical University of Silesia in Katowice, 4 Jagiellońska Str., 41-200 Sosnowiec, Poland; kmarciniec@sum.edu.pl (K.M.); ebebenek@sum.edu.pl (E.B.); mkadela@sum.edu.pl (M.K.-T.); pawel.marek.pecak@gmail.com (P.P.); boryczka@sum.edu.pl (S.B.); 2 National Medicines Institute, 30/34 Chełmska Str., 00-725 Warszawa, Poland; aleksandra_dabrowska@o2.pl

**Keywords:** triterpenes, anti-HIV, SARS-CoV-2, bevirimat

## Abstract

Lupane-type pentacyclic triterpenes such as betulin and betulinic acid play an important role in the search for new therapies that would be effective in controlling viral infections. The aim of this study was the synthesis and evaluation of in vitro anti-HIV-1 activity for phosphate derivatives of 3-carboxyacylbetulin **3**–**5** as well as an in silico study of new compounds as potential ligands of the C-terminal domain of the HIV-1 capsid–spacer peptide 1 (CA-CTD-SP1) as a molecular target of HIV-1 maturation inhibitors. In vitro studies showed that 28-diethoxyphosphoryl-3-*O*-(3′,3′-dimethylsuccinyl)betulin (compound **3**), the phosphate analog of bevirimat (betulinic acid derivative, HIV-1 maturation inhibitor), has IC_50_ (half maximal inhibitory concentration) equal to 0.02 μM. Compound **3** inhibits viral replication at a level comparable to bevirimat and is also more selective (selectivity indices = 1250 and 967, respectively). Molecular docking was used to examine the probable interaction between the phosphate derivatives of 3-carboxyacylbetulin and C-terminal domain (CTD) of the HIV-1 capsid (CA)–spacer peptide 1 (SP1) fragment of Gag protein, designated as CTD-SP1. Compared with interactions between bevirimat (**BVM**) and the protein, an increased number of strong interactions between ligand **3** and the protein, generated by the phosphate group, were observed. These compounds might have the potential to also inhibit SARS-CoV2 proteins, in as far as the intrinsically imprecise docking scores suggest.

## 1. Introduction

Despite significant advances in medicine and continuous work on new pharmacotherapy methods, in addition to common preventive vaccination programs, in the first two decades of the 21st century, the World Health Organization (WHO) recorded many epidemics of viral diseases. Among the great epidemics of this period were severe acute respiratory syndrome (SARS) in the year 2003, the influenza H1N1 pandemic in 2009, Middle East respiratory syndrome (MERS) in 2012, and the Zika virus in 2005, as well as the HIV/AIDS pandemic, which peaked in 2005–2012 [1]. The beginning of 2020 brought a new pandemic produced by the rapidly spreading virus that causes COVID-19 disease. In the case of each new pathogen, the science world faces the difficult challenge of finding an effective therapy.

Chemical modification of natural substances is an important method used to obtain promising new therapeutic agents. Pentacyclic triterpenes and their semi-synthetic derivatives are a large group of compounds known to demonstrate biological activity, including antitumor, antiviral, antimalarial, antibacterial, anti-inflammatory, and hepatoprotective effects [2]. Antiviral activity of compounds with pentacyclic triterpene structure has been reported in relation to viruses such as HIV, hepatitis virus C (HCV), various types of influenza viruses, SARS, hepatitis B virus (HBV), herpes viruses (HSV), human enterovirus 71 (EV71), and Epstein–Barr virus (EBV) [3]. The first lupane-type triterpenoid to undergo phase IIb clinical trials was 3-*O*-(3′,3′-dimethylsuccinyl)betulinic acid, known as bevirimat (BVM). Further studies on this compound were suspended due to resistance emerging among infected patients [4]. However, understanding the mechanism of its antiviral activity has opened a new direction in research on inhibitors of virus maturation [5].

Among the drugs used in the treatment of viral infections, compounds containing phosphate or the phosphonate moiety within their structure have an important place. Examples of such drugs include cidofovir (active against herpes simplex virus HSV-1 and HSV-2, as well as Epstein–Barr virus), adefovir and pradefovir (active against HBV), tenofovir (active against HIV-1, HIV-2, and HBV), and foscarnet (active against herpes viruses and HIV) [6,7,8]. Indeed, several patents describing phosphorus derivatives of triterpenes with antiviral activity can be found in the literature [9,10,11]. Anti-HIV-1 activity has also been reported for BVM analogues that contain a phosphate or phosphonate substituent in the isopropenyl moiety [12]. For example, 30-diethoxyphosphoryl-3-*O*-(3′,3′-dimethylsuccinyl)betulinic acid described in the work [12] demonstrates in vitro anti-HIV-1 activity with an IC_50_ value similar to that of BVM, and with a lower cytotoxicity. The results of molecular docking into the Gag C-terminal domain of the HIV-1 capsid–spacer peptide 1 (CA-CTD-SP1) fragment, which is the molecular target of maturation inhibitors, suggest that the potential molecular mechanism of these compounds may be the same, but is further enhanced by the introduction of a phosphonate moiety [12]. However, this is not the only mechanism of anti-HIV activity that has been described for triterpene derivatives [3].

Coronaviruses (CoVs), members of the family Coronaviridae and subfamily Coronavirinae, are enveloped positive-strand RNA viruses that have glycoprotein spikes projecting from their viral envelopes and thus exhibit a corona or halo-like appearance [13]. A new strain of CoV was identified at the end of 2019, initially named 2019-nCoV (“n” denoting “novel”), which emerged during an outbreak in Wuhan, China [14].

Research is currently underway on the use of several dozen drugs and their combinations to combat the new coronavirus. First, therapy based on drugs already in use is sought, leading to the study of antiviral therapies using corticosteroids and anti-malarial agents, as well as cellular therapies. In the treatment of people infected with SARS-CoV-2 coronavirus, in many countries, attempts are also being made to use anti-HIV drugs that may be useful in the early stages of infection [15]. 

The subject of this work is the synthesis of new betulin phosphate derivatives having carboxyacyl substituents at the C3 position and an evaluation of their activity against HIV-1. For newly synthesized compounds, molecular docking to the active sites of CA-CTD-SP1 as the molecular target of HIV maturation inhibitors was performed. Moreover, recent in silico studies indicate the possibility of using triterpenoids as substances that may interact with various SARS-CoV-2 proteins [16,17,18,19]. Following that, molecular docking of selected compounds to five proteins of SARS-CoV-2 was performed.

## 2. Materials and Methods

### 2.1. Synthesis

All solvents used in reactions were dried and purified according to standard procedures. The progress of reaction was monitored by thin-layer chromatography (TLC) (silica gel 60 254F plates, Merck, Darmstadt, Germany). The chromatographic plates were developed in a mixture of hexane and ethyl acetate (3:2, *v*/*v*) or chloroform and ethanol (15:1, *v*/*v*). The spots were visualized by spraying them with a solution of 5% sulfuric acid and heating to 100 °C. The compounds were purified by column chromatography (silica gel 60, particle size < 63 m, Merck) in the solvent system indicated.

Acylation reactions were performed in a microwave reactor (Discover SP-D, CEM Corporation, Matthews, NC, USA).

Melting points of obtained compounds were measured in open capillary tubes on an Electrothermal melting point apparatus without correction. ^1^H NMR (600 MHz), ^13^C NMR (150 MHz) and ^31^P NMR (243 MHz) spectra were recorded on a Bruker AVANCE III HD 600 spectrometer (Bruker, Billerica, MA, USA) in deuterated CDCl_3_, using the residual solvent signal as an internal standard. Chemical shifts values were reported in parts per million (ppm). Multiplicity was designated as singlet (s), doublet (d), doublet of doublets (dd) and multiplet (m). Infrared spectra (pellets, KBr, Merck, Darmstadt, Germany) were obtained using an IRAffnity-1 FTIR spectrometer (Shimadzu, Kyoto, Japan). The measurement was recorded in the range of 4000–1000 cm^–1^ at 295K. High-resolution mass spectra have been measured with Bruker Impact II (Bruker, Billerica, MA, USA). Calculation of the theoretical molecular mass for compounds was performed using “The Exact Mass Calculator, Single Isotope Version” (http://www.sisweb.com/referenc/tools/exactmass.htm; (Ringoes, NJ, USA)).

Synthesis of BVM [3-*O*-(3’,3’-dimethylsuccinyl)betulinic acid], used in the study as a reference, was performed as was previously described [12]. The final product was obtained at a yield of 70%. The melting point and spectroscopic characteristics of the compound were consistent with the literature data [20].

#### 2.1.1. Synthesis of 28-Diethoxyphosphorylbetulin **2**

To the solution of 1 mmol of betulin **1** in 3 mL tetrahydrofuran (THF), pyridine (2.6 mmol, 0.24 mL) and *N*,*N*-dimethylaminopyridine (DMAP; 0.1 mmol, 12 mg) was added. The obtained mixture was cooled in an ice-water bath to 0 °C, then diethylchlorophosphate (2 mmol, 0.29 mL) was added dropwise. The reaction was carried out under argon atmosphere for 9 h. Then, the volatile components were evaporated on a vacuum evaporator. Dichloromethane (15 mL) was added to the residue and washed with saturated sodium bicarbonate solution and water. The organic layer was dried with anhydrous sodium sulphate (VI), then concentrated until dry. The product was purified by column chromatography (SiO_2_; hexane/ethyl acetate ratio: 3:2, *v*/*v*) yielding compound **2**.

##### 28-Diethoxyphosphorylbetulin **2**

Yield 86%; m.p. 181–184 °C; R_f_ = 0.2 (hexane/ethyl acetate, 3:2, *v*/*v*); IR (KBr) ν (cm^−1^): 1739, 1263, 1034; ^1^H NMR (CDCl_3_) δ (ppm): 4.7 (m, 1H, H29), 4.61 (m, 1H, H29), 4.21 (dd, *J* = 4.2 Hz, *J* = 9.6 Hz, 1H, H28), 4.15 (m, 4H, OCH_2_CH_3_), 3.78 (dd, *J* = 4.2 Hz, *J* = 9.6 Hz, 1H, H28), 3.21 (dd, *J* = 4.8 Hz, *J* = 12.0 Hz, 1H, H3), 2.40 (m, 1H, H19), 2.05–1.90 (m, 3H, H2, H12, H15), 1.75–1.50 (m, 11H, H1, H2, H6, H16, H18, H21, H22), 1.69 (s, 3H, 30-CH_3_), 1.45–1.36 (m, 5H, H7, H9, H15), 1.37 (m, 6H, OCH_2_CH_3_), 1.29–1.10 (m, 3H, H11, H12), 1.10 (m, 1H, H13), 1.04 (s, 3H, CH_3_), 0.99 (s, 3H, CH_3_), 0.99 (s, 3H, CH_3_), 0.91 (m, 1H, H1), 0.84 (s, 3H, CH_3_), 0.78 (s, 3H, CH_3_), 0.70 (m, 1H, H5); ^13^C NMR (CDCl_3_) δ (ppm): 150.1 (C20), 109.9 (C29), 79.0 (C3), 66.1 (C28), 63.7, 55.3, 50.4, 48.6, 47.7, 47.2, 47.2, 40.9, 38.9, 38.7, 37.6, 37.2, 34.2, 34.1, 29.5, 29.2, 28.0, 27.4, 26.9, 25.2, 20.8, 19.1 (C30), 18.3, 16.3, 16.2, 16.1, 16.0, 15.4, 14.8; ^31^P NMR (CDCl3) δ (ppm): −0.18. HR-MS (APCI) *m*/*z*: C_34_H_58_O_5_P [(M–H)^−^], Calc. 577.4022; Found 577.4011.

#### 2.1.2. General Method of Synthesis 3-Carboxyacyl Derivatives **3–5**

To the solution of 1 mmol 28-diethoxyphosphorylbetulin **2** in 2 mL pyridine, DMAP (1.5 mmol, 0.19 g) and 5 mmol of appropriate acid anhydride (2,2-dimethylsuccinic anhydride or 3,3-dimethylglutaric anhydride or 2,2-dimethylglutaric anhydride) was added. The reaction vessel was placed in a microwave reactor and the reaction was carried out for 1.5 h at a temperature of 130 °C at a maximum wave power (300 W). After cooling, the mixture was diluted with 25 mL of ethyl acetate, and then washed with a 20% hydrochloric acid solution and with water. The organic layer was dried with anhydrous sodium sulphate (VI) and concentrated until dry in a vacuum evaporator. The crude product was purified by column chromatography (SiO_2_; chloroform/ethanol ratio: 15:1, *v*/*v*) to obtain phosphorus derivatives of 3-carboxyacyl-28-diethoxyphosphorylbetulin **3**–**5**.

##### 28-Diethoxyphosphoryl-3-O-(3′,3′-dimethylsuccinyl)betulin **3**

Yield 42%; m.p. 94–96 °C; R_f_ = 0.42 (chloroform/ethanol, 15:1, *v*/*v*); IR (KBr) ν (cm^−1^): 1739, 1263, 1034; ^1^H NMR (CDCl_3_) δ (ppm): 4.7 (m, 1H, H29), 4.61 (m, 1H, H29), 4.51 (dd, *J =* 4.8 Hz, *J =* 11.4 Hz, 1H, H3), 4.21 (m, 1H, H28), 4.16 (m, 4H, OCH_2_CH_3_), 3.77 (m, 1H, H28), 2.69 (d, *J =* 15.6 Hz, 1H, CH_2_-2′, dimethylsuccinic), 2.60 (d, *J =* 15.6 Hz, 1H, CH_2_-2′, dimethylsuccinic), 2.40 (m, 1H, H19), 2.01–1.91 (m, 3H, H2, H12, H15), 1.73–1.61 (m, 9H, H1, H2, H16, H18, H21, H22), 1.7 (s, 3H, 30-CH_3_), 1.52 (m, 2H, H6), 1.43–1.2 (m, 8H, H7, H9, H11, H12, H13, H15), 1.36 (m, 6H, OCH_2_CH_3_), 1.32 (s, 3H, 3′-CH_3_), 1.28 (s, 3H, 3′-CH_3_), 1.05 (s, 3H, 26-CH_3_), 1.00 (s, 3H, 27-CH_3_), 0.91 (m, 1H, H1), 0.86 (s, 6H, 23-CH_3_, 25-CH_3_), 0.83 (s, 3H, 24-CH_3_), 0.79 (m, 1H, H5); ^13^C NMR (CDCl_3_) δ (ppm): 181.2 (COOH), 171.1 (COO), 150.0 (C20), 110.0 (C29), 81.5 (C3), 66.1 (C28), 63.8 (POCH_2_), 63.8 (POCH_2_), 55.4 (C5), 50.2 (C9), 48.6 (C18), 47.7 (C19), 47.2 (C17), 44.7 (C2′), 42.7 (C14), 40.9 (C8), 40.4 (C3′), 38.4 (C1), 37.7 (C4), 37.1 (C13), 37.0 (C10), 34.1 (C7), 31.9 (C22), 29.7 (C21), 29.4 (C15), 29.1 (C16), 28.0 (C23), 26.8 (C12), 25.6 (CH_3,_ dimethylsuccinic)_,_ 25.0 (CH_3_, dimethylsuccinic), 23.6 (C2), 20.8 (C11), 19.1 (C30), 18.2 (C6), 16.5 (C24), 16.2 (POCH_2_CH_3_), 16.2 (POCH_2_CH_3_), 16.1 (C26), 16.0 (C25), 14.8 (C27); ^31^P NMR (CDCl_3_) δ (ppm): −0.22. HR-MS (APCI) *m*/*z*: C_40_H_66_O_8_P [(M–H)^−^], Calc. 705.4495; Found 705.4489.

##### 28-Diethoxyphosphoryl-3-O-(3′,3′-dimethylglutaryl)betulin **4**

Yield 37%; m.p. 140–143 °C; R_f_ = 0.38 (chloroform/ethanol, 15:1, *v*/*v*); IR (KBr) ν (cm^−1^): 2949, 1734, 1247, 1029; ^1^H NMR (CDCl_3_) δ (ppm): 4.7 (m, 1H, H29), 4.61 (m, 1H, H29), 4.52 (dd, *J =* 4.8 Hz, *J =* 11.4 Hz, 1H, H3), 4.21 (m, 1H, H28), 4.16 (m, 4H, OCH_2_CH_3_), 3.76 (m, 1H, H28), 2.49–2.45 (m, 3H, CH_2_, dimethylglutaric and H19), 2.41 (m, 2H, CH_2_, dimethylglutaric); 2.02–1.91 (m, 3H, H2, H12, H15), 1.75–1.61 (m, 9H, H1, H2, H16, H18, H21, H22), 1.54 (m, 2H, H6), 1.70 (s, 3H, 30-CH_3_), 1.45–1.17 (m, 7H, H7, H9, H11, H12, H15), 1.37 (m, 6H, OCH_2_CH_3_), 1.16 (s, 6H, 2× CH_3_), 1.10 (m, 1H, H13), 1.05 (s, 3H, CH_3_), 0.99 (s, 3H, CH_3_), 0.91 (m, 1H, H1), 0.88 (s, 3H, CH_3_), 0.86 (s, 3H, CH_3_), 0.86 (s, 3H, CH_3_), 0.80 (m, 1H, H5); ^13^C NMR (CDCl_3_) δ (ppm): 174.4 (COOH), 173.0 (COO), 150.1 (C20), 110.0 (C29), 81.0 (C3), 66.1 (C28), 63.8 (POCH_2_), 63.8 (POCH_2_), 55.4, 50.2, 48.6, 47.7, 47.2, 45.6 (C2′), 45.3 (C4′), 45.1, 42.7, 40.9, 38.3, 37.7, 37.6, 37.0, 34.1, 32.9 (C3′), 31.0, 29.1, 28.2 (CH_3,_ dimethylglutaric), 28.1 (CH_3,_ dimethylglutaric), 28.0, 26.8, 25.1, 23.8, 20.8, 19.1 (C30), 18.2, 16.6, 16.3, 16.2, 16.1, 16.0, 14.8; ^31^P NMR (CDCl_3_) δ (ppm): −0.20. HR-MS (APCI) *m*/*z*: C_41_H_68_O_8_P [(M–H)^−^], Calc. 719.4652; Found 719.4643.

##### 28-Diethoxyphosphoryl-3-O-(4′,4′-dimethylglutaryl)betulin **5**

Yield 38%; m.p. 96–98 °C; R_f_ = 0.41 (chloroform/ethanol, 15:1, *v*/*v*); IR (KBr) ν (cm^−1^): 2947, 1732, 1259, 1028; ^1^H NMR (CDCl_3_) δ (ppm): 4.70 (m, 1H, H29), 4.61 (m, 1H, H29), 4.49 (dd, *J =* 5.4 Hz, *J =* 10.8 Hz, 1H, H3), 4.21 (m, 1H, H28), 4.16 (m, 4H, OCH_2_CH_3_), 3.75 (m, 1H, H28), 2.40 (m, 1H, H19), 2.40 (m, 1H, H19), 2.36 (m, 2H, CH_2_, dimethylglutaric); 2.00–1.91 (m, 3H, H2, H12, H15), 1.91 (m, 2H, CH_2_, dimethylglutaric), 1.75–1.58 (m, 9H, H1, H2, H16, H18, H21, H22), 1.54 (m, 2H, H6), 1.70 (s, 3H, 30-CH_3_), 1.45–1.17 (m, 7H, H7, H9, H11, H12, H15), 1.38 (m, 6H, OCH_2_CH_3_), 1.24 (s, 6H, 2× CH_3_), 1.08 (m, 1H, H13), 1.05 (s, 3H, CH_3_), 0.99 (s, 3H, CH_3_), 0.91 (m, 1H, H1), 0.86 (s, 6H, 2× CH_3_), 0.85 (s, 3H, CH_3_), 0.80 (m, 1H, H5); ^13^C NMR (CDCl_3_) δ (ppm): 181.8 (COOH), 173.2 (COO), 150.0 (C20), 110.0 (C29), 81.0 (C3), 66.1 (C28), 63.8 (POCH_2_), 63.8 (POCH_2_), 55.3, 50.2, 48.6, 47.8, 47.2, 42.7, 41.5, 40.9, 38.3, 37.9, 37.6, 37.1, 37.0, 35.0, 34.1, 30.6, 29.4, 29.1, 28.0 (CH_3,_ dimethylglutaric), 26.8, 25.1, 25.0, 24.9, 23.7, 20.8, 19.1 (C30), 18.2, 16.6, 16.2, 16.2, 16.1, 16.0, 14.8; ^31^P NMR (CDCl_3_) δ (ppm): −0.21. HR-MS (APCI) *m*/*z*: C_41_H_68_O_8_P [(M–H)^−^], Calc. 719.4652; Found 719.4645.

### 2.2. Biological Activity

#### 2.2.1. Cytotoxicity

For the determination of compounds’ cytotoxicity, CEM-T4 cells were obtained from the NIH AIDS Reagent Program (NIH, US) and were cultured in RPMI supplemented with 10% FCS (Biochrom) and antibiotics at 37 °C in 5% CO_2_ on 96-well culture plates. The experiments were carried out in media containing tested compounds in concentrations of the appropriate range. Cultures in a neat medium (RPMI, 10% FCS) were used as a control. Viability of cells was determined after 7 days using the MTT assay [21] in which 10 μL of MTT solution (5 mg/mL) was added to each culture plate well, and cultures were incubated for 3 h at a temperature of 37 °C. After the centrifugation, the supernatant was removed, and DMSO was added for lysis of the cells and to dissolve crystals of formazan. Color intensity was measured with a plate reader at 560 nm.

#### 2.2.2. Anti-HIV Activity

CEM-T4 cells were preincubated (culture plates with 96 flat bottom wells) for 24 h under standard conditions (37 °C, 5% CO_2_) and in a standard medium (RPMI, FCS 10%) enriched with tested compounds in the concentration range from 0.02 to 10 μM. In each well, 20,000 cells were suspended in the solution of a tested compound (200 μL). For each concentration, cultures were run in triplicate. A wild-type HIV-1 was isolated from the HIV-positive patient in the Laboratory of Virology of the National Medicines Institute (Warsaw, Poland) and was used as a reference. A culture of CEM-T4 lines in a standard neat medium (RPMU, FCS 10%) was used to produce viruses. After 24 h of incubation in a medium enriched with a tested compound, cells were inoculated with a known amount of HIV, and after 7 days, HIV replication was evaluated through the measurement of secreted viral protein p24 carried out with the enzyme-linked immunosorbent assay (ELISA) technique [22].

For each tested compound and for each concentration, the measurements of p24 antigen were done in triplicate using the Genscreen ULTRA HIV Ag-Ab Kit (Biorad, Warszawa, Poland) and following manufacturer’s instructions.

### 2.3. Molecular Docking Study

The 3D structures of studied compounds were generated in their low-energy conformation using the Gaussian 16 (revision A.03) computer code [23] with the density functional theory (DFT, B3LYP) and 63–11 + G(d,p) basis sets. Target macromolecules for molecular docking studies were obtained from the Protein Data Bank (https://www.rcsb.org/; Research Collaboratory for Structural Bioinformatics, USA). We used crystal structure of the CA-CTD-SP1 fragment of HIV-1 Gag, SARS-CoV-2 main protein, SARS-CoV-2 RNA-dependent RNA polymerase, and SARS-CoV-2 spike ectodomain (PDB IDs: 5I4T, 5R7Z, 6M71, and 6VYB respectively). Incomplete or missing side chains of CA-CTD-SP1 protein were restored with the SWISS-MODEL Workspace (https://swissmodel.expasy.org/; Swiss Institute of Bioinformatics, Basel, Switzerland) [24]. Geometry optimization of the model was achieved by the optimization protocol in YASARA Energy Minimization Server (http://www.yasara.org/-minimizationserver.htm; YASARA Biosciences GmbH, Vienna, Austria). The SARS-CoV-2 main protease (M^pro^) structure was obtained from PDB (PDB ID: 5R7Z). The native ligand for 5R7Z is ***N*-[2-(5-fluoro-1*H*-indol-3-yl)ethyl]ethanamide (PDB ID: HWH)**. The region of interest used for Vina docking was defined as all M^pro^ residues within 40 × 40 × 40 Å of the reference ligand. The homology model of the SARS-CoV-2 E protein catalytic domain was also generated in the SWISS-MODEL workspace [24]. Based on maximum sequence identity (95%), structure with PDB ID 5X29 is detected as the best homologous structure of human SARS-CoV-2 E protein. Geometry optimization of model was done by the optimization protocol in YASARA. For toxic/side effect study we used crystal structure of the pyruvate kinase (PDB ID: 6NU1), aconitase (PDB ID: 1C96), cytochrome c (PDB ID: 1HRC), carbamoyl phosphate synthetase 1 (PDB ID: 5DOU), hypoxanthine-guanine phosphoribosyltransferase (PDB ID: 6AR9), glutamate dehydrogenase (PDB ID: 1BVG).

In this study, AutoDock Vina [25] tool compiled in PyRx [26] was employed to perform molecular docking. AutoDock Vina incorporates limited flexibility in the receptor, and it combines an empirical free-energy force field with a Lamarckian Genetic Algorithm, providing fast prediction of bound conformations with predicted free energies of association. The volume was set as 40 × 40 × 40 Å. The region of interest used for AutoDock Vina docking was fixed as X = 0.041, Y = 0.010 and Z = 3.855 for HIV-1 Gag and X = −27.585, Y = 2.501 and Z = 28.139 for SARS-CoV-2 M^pro^, X = 114,416, Y = 122.286 and Z = 139.457 for SARS-CoV-2 RdRp, X = −0.496, Y = 0.00 and Z = 0.00 for SARS-CoV-2 E protein and X = 177,973, Y = 198.333, Z = 227.722 Å for SARS-CoV-2 S protein, X = 42.156, Y = −15.872 and Z = 28.523 for pyruvate kinase, X = 69.590, Y = 67.860 and Z = 68.790 for aconitase, X = 46.737, Y = 23.218 and Z = 5.557 for cytochrome c, X = −29.871, Y = −33.177, Z = 51.502 for carbamoyl phosphate synthetase 1, X = −2.844, Y = −22.675 and Z = −43.271 for hypoxanthine-guanine phosphoribosyltransferase and X = −17.201, Y = 20.346, Z = 22.393 Å for glutamate dehydrogenase. After calculations, only the 9 highest-scored poses were returned as a docking result for ligand–cavity configuration. All the obtained results were ranked according to their score values and presented in kcal/mol. Molecular docking details were visualized using the BIOVIA Discovery Studio virtual environment [27].

### 2.4. Molecular Dynamics Simulations

Appropriate AutoDock Vina output complexes were prepared for simulations using QwikMD [28] built in Visual Molecular Dynamics (VMD https://www.ks.uiuc.edu/Research/vmd/; Theoretical and Computational Biophysics Group, University of Illinois at Urbana-Champaign, Illinois, USA) software ver 1.9.3 [29]. Simulation was carried out with NAMD (https://www.ks.uiuc.edu/Research/namd/; Theoretical and Computational Biophysics Group, University of Illinois at Urbana-Champaign, Illinois, USA) ver. 2.13 [30] using CHARMM27 force field. Periodic boundary conditions with an explicit solvent were employed. Parameterization of ligands was conducted using a CGenFF server [31,32]. All protein and protein–ligand systems have been solvated with TIP3P cubic water box at 15 Å thickness. To neutralize the system, 0.15 mol/L of NaCl salt was added. Simulation protocol consists of 2000 steps of minimization, 144,000 steps of annealing, 500,000 steps of equilibration and 5,000,000 steps (10ns) of production MD simulation. The system was heated to 300 K at rate of one Kelvin degree per 1 ps. Langevin dynamics were used to control the temperature. Minimization, annealing and equilibration were restrained to backbone atoms of the protein, while the production run was unrestrained. Timestep was set to 2 fs, all runs were performed in NPT ensemble. VMD was used to analyze the results.

## 3. Results and Discussion

### 3.1. Chemistry

**Scheme 1:**
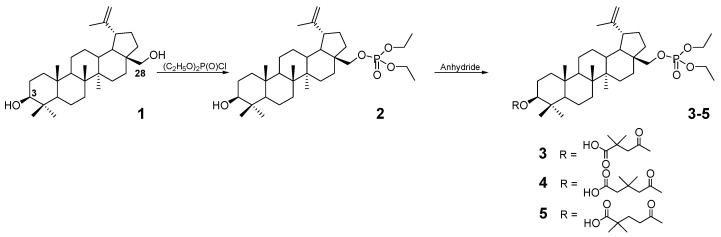
Synthesis of phosphate derivatives of 3-carboxyacylbetulin **3**–**5**.

The above-mentioned literature data and results of the anti-HIV activity test obtained for phosphate and phosphonate derivatives of betulinic acid [12] have become the starting point for the synthesis of betulin phosphate derivatives. The obtained compounds have a carboxyacyl group in position C3, whose presence is important for action against HIV-1, and a diethyl phosphate group in position C28 (Scheme 1). This work attempts to answer the question of how the introduction of a phosphate substituent affects the activity of synthesized compounds, compared to substances with known potential—in this case, **BVM**.

Betulin **1** obtained from birch bark harvested in southern Poland by extraction with dichloromethane was used as the starting substrate. After concentrating the extracts, the produced substance was purified by crystallization from ethanol. In the first stage, a phosphorylation reaction of a hydroxyl group at the C28 position was carried out using diethyl chlorophosphate. After purification, 28-diethoxyphosphorylbetulin **2** was obtained at a yield of 86%. Compound **2** was then reacted with 3′,3′-dimethylsuccinic, 4′,4′-dimethylglutaric, and 3′,3′-dimethylglutaric anhydride according to the method previously described [33]. Derivatives **3**–**5** were obtained at a yield of 32−38%.

### 3.2. Biological Activity

Newly synthesized compounds **2**–**5** were evaluated for their activity against HIV-1 in a CEM-T4 cell line. First, a cytotoxicity assessment of the synthesized derivatives was performed using the MTT [3-(4,5-dimethylthiazol-2-yl)-2,5-diphenyltetrazolium bromide]. The results were expressed as CC_50_ (concentration of compound causing 50% cell death). Anti-HIV activity was expressed as IC_50_ (concentration of compound causing 50% inhibition of replication) and consisted of measurements of p24 antigen made using the Genscreen ULTRA HIV Ag-Ab Kit. Betulin **1** and **BVM** were used as reference compounds. The bioassay results are presented in Table 1.

As can be seen, betulin **1** and betulin phosphate **2** did not demonstrate the ability to inhibit HIV replication in the tested range of concentrations. Betulin derivatives containing a 3′,3′-dimethylglutaric group that exhibit significant activity against HIV-1 are described in the literature [34,35]. Corresponding phosphorus derivative **4** demonstrated good activity and selectivity that were higher than obtained for 4′,4′-dimethylglutaric derivative **5**. On the other hand, compound **4** was more active than the previously described 3′,3′-dimethylglutaric derivatives of betulinic acid, which in the C30 position contained a diethylphosphate or diethylphosphonate group (IC_50_ = 0.6 μM and 0.9 μM, respectively) [12].

The results obtained for phosphate derivatives of 3-carboxyacylbetulin **3**–**5** indicate that the most active compound is derivative **3** with R = HOC(O)C(CH_3_)_2_CH_2_C(O), which shows activity comparable to that of **BVM** (IC_50_ = 0.02 and 0.03 µM, respectively). A chart of changes in HIV-1 inhibition of 3-carboxyacylbetulin phosphate and **BVM** in the tested concentration range is attached to the Supplementary Materials (Figure S1). However, compound **3** has a higher selectivity for action (therapeutic index (TI) = 1250 and 967, respectively). Among the tested phosphate derivatives, compound **3** showed the lowest cytotoxicity, but it was at a level comparable to BVM. Other phosphate derivatives were more toxic and also less active against HIV. As results from the data presented in the Table 1, compounds with a longer carbon chain in the carboxyacylic substituent are more cytotoxic. Dimethylglutaric derivatives have an IC_50_ of 19.68 µM and 15.11 µM for **4** and **5**, respectively. The IC_50_ value determined for the dimethylsuccinic derivative **3** is 25.36 µM and for **BVM** is 29.02 µM. The position of the methyl groups on the substituent chain at C3 does not affect the cytotoxicity of the compound. A chart of changes in cytotoxicity of new phosphate derivatives **2**–**5** in the tested concentration range is attached to the Supplementary Materials (Figure S2).

An important issue in the study of new chemical substances considered as potential drug candidates is the analysis of their physicochemical parameters. The in silico pharmacokinetic study is being used in the development of new drugs with good oral bioavailability, low toxicity and the least side effects. For phosphate derivatives **2**–**5** the ADME (Adsorption-Distribution-Metabolism-Excretion) properties (Supplementary Materials Table S1) such as number of hydrogen bond acceptors (nHBA), number of hydrogen bond donors (nHBD), lipophilicity (Log P), molecular weight M, number of rotatable bonds (nROTB) and topological polar surface area (TPSA) were evaluated through the pkCSM online server (http://biosig.unimelb.edu.au/pkcsm/; University of Melbourne, Australia) [36].

The hydrophobicity of compounds was determined on the basis of Log P values. The Log P parameter determines the strength of the drug and its distribution in the body after absorption stage. The obtained Log P values (9.20–10.65) of triterpene derivatives indicate poor permeability across the cell membrane. The compounds shown in the Table S1 having a number of rotational bonds in the range 8–13 show high conformational flexibility and good binding affinity with the binding pocket. Topological polar surface area (TPSA) is important criteria for the determination of oral bioavailability. Compounds that meet the criterion that TPSA ≤ 140 Å may show oral bioavailability [37,38].

The values of the parameters log PS, log BB and TPSA for the tested derivatives are characteristic for the CNS-nonactive drugs [36].

### 3.3. Molecular Docking

The tested compounds can exist as two chemical species, carboxylic acids or carboxylate salts, depending on the pH of the aqueous solution. Calculations performed with ACD/Percepta software [39] for **BVM** and compounds **3**–**5** show that in all cases the content of the carboxylate states at physiological pH is 100%. Therefore, the carboxylate states of betulin derivatives were used in docking calculations. The three-dimensional (3D) structures of ligands required for docking studies were generated in their low-energy conformation using Gaussian 16 computer code [24].

**Figure 1:**
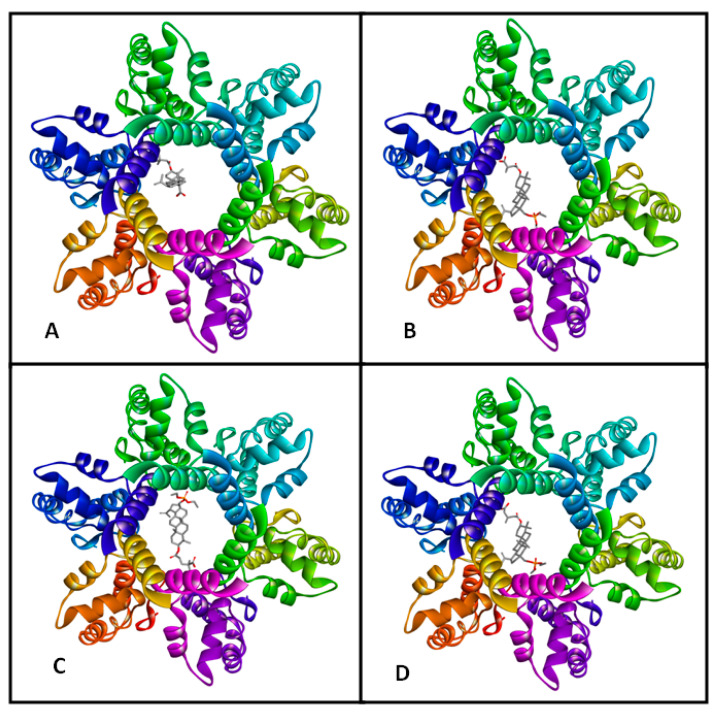
Predicted binding model of bevirimat (**BVM**) (**A**) and compounds **3**−**5** ((**B−D**), respectively) with HIV-1 capsid (CA) C-terminal domain (CTD)-spacer peptide 1 (SP1) protein.

The last step of HIV-1 replication occurs after release of immature virion outside host cell. During maturation process, viral protease cleaves Gag precursor protein to individual matrix (MA), spacer peptide (SP1), capsid (CA) and nucleocapsid (NC) peptides. This step is essential to produce mature and infectious virions. Recent studies report that the immature CA-CTD-SP1 assembles to create a hexameric structure which forms a cone-shaped core built up with multiple hexameric proteins. Protease can access the cleavage site only after the six-helix bundle unfolds [40]. Based on atomic coordinates of the CTD of CA and SP1 of HIV-1 Gag deposited in the Protein Data Bank (PBD ID 5I4T), one active site cavity was predicted [41]. This cavity was located inside the pore formed by the six-helix SP1 bundle (Figure 1).

We would like to point out that we used AutoDock ver.4.2.6 in our previous research on the antiviral activity of betulinic acid derivatives [12]. In the present study, we decided to use the program AutoDock Vina (referred to as Vina) for in silico research. Vina uses a sophisticated gradient optimization method in its local optimization procedure. The calculation of the gradient effectively gives the optimization algorithm a “sense of direction” from a single evaluation. Evaluation of the speed and accuracy of Vina during flexible docking showed an improvement in speed of approximately two orders of magnitude, and a significantly higher accuracy of the binding mode prediction compared to AutoDock [25]. We also decided to redock the **BVM** molecule to HIV-1 CA-CTD-SP1 with the use of Vina software.

The betulin derivatives ranked by AutoDock Vina are shown in Table 2. The lowest scores for binding energy (kcal/mol) of protein−ligand complexes correspond to a strong binding affinity, and the most probable ligand−protein system in vivo.

Results obtained with Vina indicate that compound **3** exhibits a lower binding energy compared to the reference **BVM** (Table 2).

**Figure 2:**
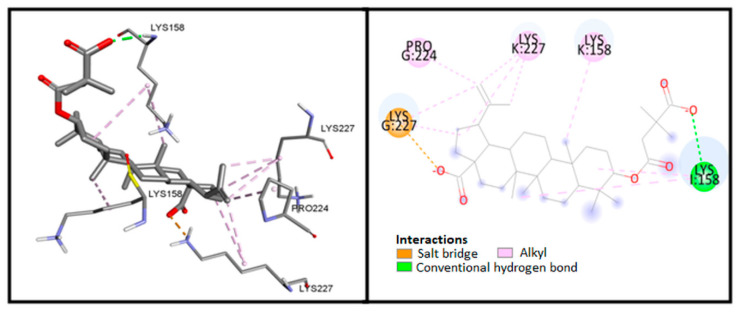
Visualization of interaction between **BVM** and CA-CTD-SP1.

Analysis of the **BVM** complex (Figure 2) included calculations, distance measurements, and pose geometries that determined salt bridge interaction of the ligand pose with Lys227 residue of chain G and hydrogen bonding with Lys158 in chain L. In addition, numerous hydrophobic interactions influence the increased stability of the complex.

**Figure 3:**
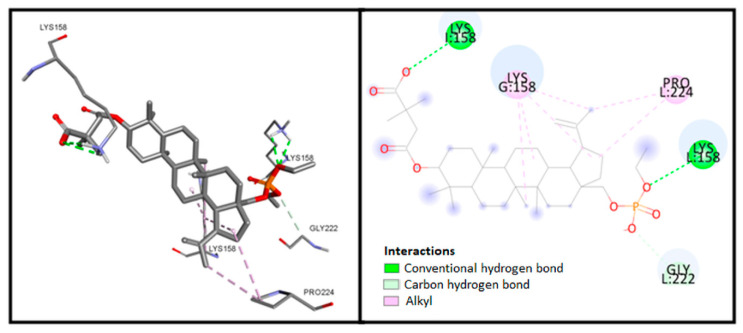
Visualization of interaction between compound **3** and CA-CTD-SP1.

Analysis of the compound **3** complex, the most potent compound in vitro (Figure 3), determined salt hydrogen bonding between the carboxylate group of the ligand and Lys158 residue of chain I, and hydrogen bonding with Lys158 in chain L and the phosphate group. In addition, numerous hydrophobic interactions, including carbon−hydrogen bonding, are also visible.

**Figure 4:**
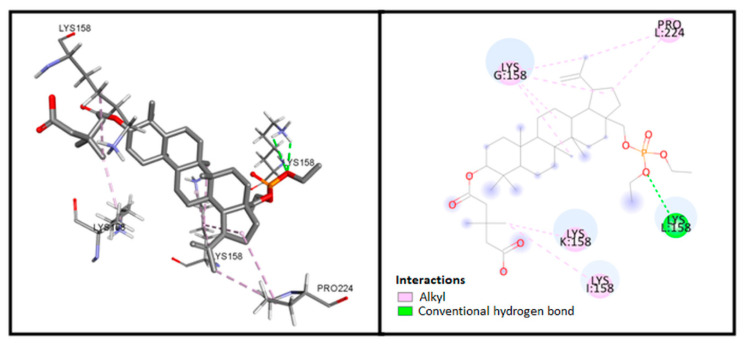
Visualization of interactions between compound **4** and CA-CTD-SP1.

According to the docking poses shown in Figure 4, the phosphate group of compound **4** can interact to form a hydrogen bond with residues Lys158 in chain L of the protein. In addition, numerous hydrophobic interactions increase the stability of the complex.

**Figure 5:**
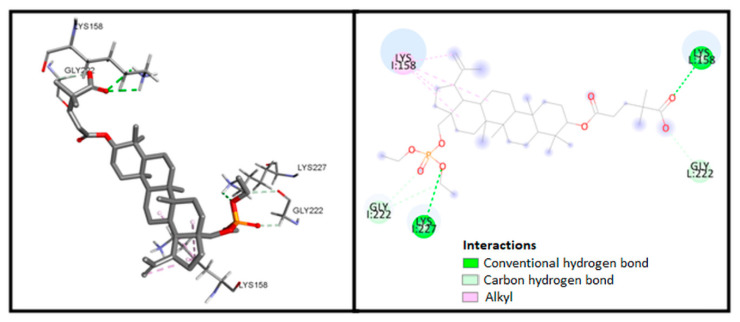
Visualization of interaction between compound **5** and CA-CTD-SP1.

Figure 5 presents the possible interaction of compound **5** inside the binding pocket of CA-CTD-SP1 after 2D analysis using the Discovery Studio Visualizer. Corresponding amino acids that are significantly involved in the interactions are as follows: Lys158 and Lys227 (hydrogen bonds); Gly222 (carbon−hydrogen bonds), and Lys 158 (alkyl–alkyl interactions).

**Figure 6:**
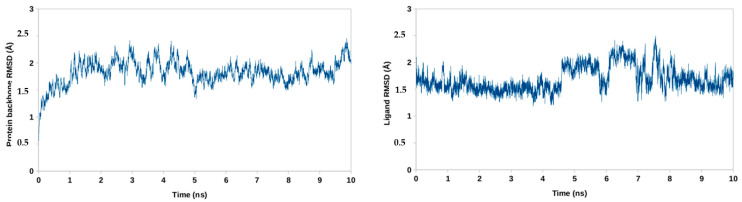
Root mean square deviation RMSD for atoms of CA-CTD-SP1 backbone (left) and **BVM** (right).

In order to validate docking results a molecular dynamics simulation (MD) has been performed. After a 10-ns run, a root mean square deviation (RMSD) of the atomic positions of ligand–protein complexes have been calculated. Low value of ligand RMSD followed by relatively constant value of protein backbone RMSD indicate complex stability and validate docking protocol. MD simulation has been performed for compound **3**, possessing the lowest score for binding energy. Both ligand and protein showed low RMSD values below 2 Å, proving stability of the ligand–protein system (Figure 6).

The docking results are in line with cytotoxicity binding assay findings, which means that the tested compounds can interact with the CA-CTD-SP1 protein.

In vitro studies in human cells, as well as preclinical studies, suggest that bevirimat should have low potential for cytotoxicity. There is no evidence of reproductive or developmental toxicity [42]. In order to check the potential toxic properties of the compounds **3**–**5**, docking study of phosphate betulin derivatives to cellular proteins was carried out.

It is known that deficiency and inhibition of some proteins important in normal cellular function may result in toxicity or side effects. Examples of these proteins are those involved in key cellular metabolism processes such as glycolytic pathway, amino acid and nucleotide metabolism, urea cycle, citric acid cycle and oxidative phosphorylation in mitochondria [43].

Table 3 gives a list of selected cellular proteins that are known to be associated with potential toxicity and side effects. Information about the physiological function, site of action and effect of deficiency/inhibition for these proteins is also given in Table 3 [44,45,46,47,48,49].

The results of docking experiments between **BVM**, compounds **3**–**5** and selected cellular proteins are shown in Table 4. According to the results of docking for the target proteins, all tested compounds showed a lower degree of fit to tested proteins compared to **BVM**. These results may indicate that modification of the bevirimat molecule by substitution of the carboxyl group by a phosphate substituent probably does not increase toxicity of phosphate derivatives compared to **BVM**.

Due to 2020’s COVID-19 pandemic, another group of RNA viruses—coronaviruses—have become interesting to scientists. The majority of its large genome is transcribed and translated into polypeptide encoding proteins. These proteins are important for gene expression. The main protease (M^pro^), and RNA-dependent RNA polymerase (RdRp) are key enzymes for coronavirus replication [50]. The M^pro^ mediates the maturation of non-structural proteins (Nsps), essential in the life cycle of the virus [51]. In research on various coronaviruses inhibitors, Nsps and its RdRp domains have been used as a promising target for new drug candidates [52].

The SARS-CoV E protein is an integral membrane. Most of the phases of the virus life cycle, such as envelope pathogenesis, formation, budding, and assembly are dependent on this protein [53,54]. It has been suggested by several studies that the absence of the SARS-COV-2 E protein may result in an “attenuated virus” [54].

Spike (S) is the fundamental protein of the coronavirus, and forms a characteristic corolla structure on the membrane of the virion [55]. Structural integrity of spike and cleavage activation play a key role in virus invasion and virulence. Therefore, blocking coronavirus form entering host cell by targeting specific receptors on the host surface, such as S protein, might be therapeutic strategy of great value for the development of the anti-viral agents [56].

**Figure 7:**
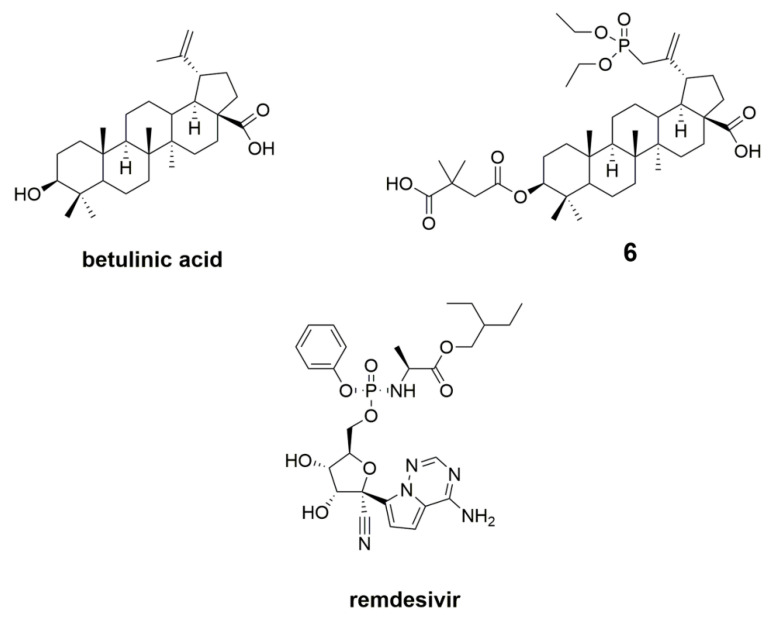
Additional ligands used for docking study for SARS-CoV-2.

No specific medicine or treatment is currently available for SARS-CoV-2-related diseases. Studies of remdesivir, phosphoramidate of an adenosine C-nucleoside analog, have brought attention to the possible application of this molecule as an anti-SARS-CoV-2 agent (Figure 7) [57]. This RdRp inhibitor [18] can inhibit the virus by inhibiting synthesis of viral nucleic acid, and has been recently authorized for emergency use in acute COVID-19 patients.

On 31 January 2020, the New England Journal of Medicine reported the diagnosis and treatment of the first SARS-CoV-2 patient in the United States [58], and remdesivir exhibited some potential in the treatment of this first patient.

Accordingly, we used remdesivir as a reference ligand. Moreover, based on our previous study, we decided to dock to SARS-CoV-2 proteins betulinic acid and 30-diethoxyphosphoryl-3-*O*-(3′,3′-dimethylsuccinyl)betulinic acid (compound **6**, Figure 7). Compound **6** demonstrated in vitro anti-HIV-1 activity with an IC_50_ value similar to that of **BVM** (IC_50_ = 0.02 µM and 0.03 µM for **6** and **BVM**, respectively), and with a lower cytotoxicity (CC_50_ = 68 µM and 29 µM for **6** and **BVM**, respectively) [12].

According to the results of docking (Table S1) obtained from AutoDock Vina, four potential SARS-CoV-2 inhibitors (**BVM**, betulinic acid, and compounds **4** and **6)** were selected based on a lower negative dock energy value. Detailed interactions between ligands and selected SARS-CoV-2 proteins are included in Supplementary Materials (Figures S3–S6 and Tables S2 and S3). MD simulation has been performed for compounds possessing the lowest score for binding energy (**BVM**, betulinic acid, and compounds **4** and **6**). Low fluctuations of RMSD of all proteins indicate that they reached stable conformation (Supplementary Materials Figures S7–S9).

## 4. Conclusions

The new betulin phosphate derivatives **2**−**5** reported in this paper were obtained from easily available material, botulin **1**, via a brief two-step synthesis. Compounds **3**−**5**, with different carboxyacylic substituents at the C3 position, exhibited in vitro anti-HIV activity with IC_50_ values in the range of 0.02−0.22 μM. Derivative **3**, which has a 3’,3’-dimethylsuccinyl moiety at the C3 position, exhibits the highest anti-HIV-1 activity (IC_50_ = 0.02 μM). For BVM, a known maturation inhibitor, the IC_50_ value determined in this study was comparable and equal to 0.03 μM. Derivative **3** was characterized by slightly higher selectivity (TI values of 1250 and 967 for **3** and BVM, respectively). Considering the literature reports on various potential mechanisms of anti-HIV activity of triterpenes, molecular modeling with CA-CTD-SP1 has been carried out. The optimal fit was demonstrated by compound **3**. This effect suggests a potential molecular target that determines anti-HIV activity of the studied compounds.

## Figures and Tables

**Table 1:** Anti-human immunodeficiency virus (HIV)-1 replication activities in human immunodeficiency virus (HIV)-infected CEM-T4 cell line.

	Compound	CC_50_ (µM)	IC_50_ (µM)	TI
**1**	Betulin	46.01 ± 3.01	NO	--
**2**	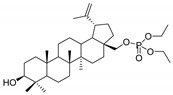	18.28 ± 1.64	NO	--
**3**	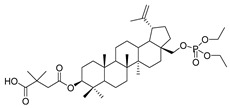	25.36 ± 1.54	0.02 ± 0.01	1250
**4**	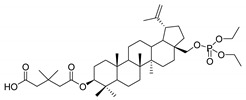	19.68 ± 1.64	0.04 ± 0.01	500
**5**	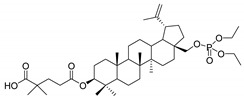	15.11 ± 1.27	0.22 ± 0.05	68
**BVM**	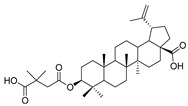	29.02 ± 2.43	0.03 ± 0.009	967

Therapeutic index (TI), the ratio of CC_50_ to IC_50._ NO—not observed.

**Table 2:** Scoring functions of tested compounds (HIV-1 protein).

Compound	HIV-1 ΔG [kcal/mol]
	CA-CTD-SP1
**3**	−6.8
**4**	−6.5
**5**	−6.2
**BVM**	−6.5

**Table 3:** Toxicity and side effect-causing protein target of drugs.

Protein	Physiological Function	Site of Action	Effect of Deficiency/Inhibition	Ref.
pyruvate kinase	glycolysis	cytosol	hemolytic anemia	[44]
aconitase	citric acid cycle	mitochondria	neurotoxicity	[45]
cytochrome c	oxidative phosphorylation	mitochondria	increased sensitivity to cell heath signals triggered by TNF-α	[46]
carbamoyl phosphate synthetase I	urea cycle	mitochondria	hyperammonemia	[47]
hypoxanthine-guanine phosphoribosyltransferase	nucleotide biosynthesis	mitochondria	hyperuricemia	[48]
glutamate dehydrogenase	amino acid degradation	mitochondria	nephrotoxicity	[49]

**Table 4:** Scoring functions of the tested compounds and cellular proteins.

Compd	Cellular Proteins ΔG [kcal/mol]					
	Pyruvate Kinase	Aconitase	Cytochrome c	Carbamoyl Phosphate Synthetase I	Hypoxanthine-Guanine Phosphoribosyltransferase	Glutamate Dehydrogenase
**BVM**	−7.4	−8.0	−4.6	−7.7	−8.1	−9.9
**3**	−6.3	−7.9	−3.3	−7.0	−7.4	−8.3
**4**	−5.8	−7.4	−4.5	−6.5	−7.5	−8.3
**5**	−6.3	−6.7	−4.5	−6.8	−7.8	−8.6

## Supplementary Materials

The following are available online at https://www.mdpi.com/2218-273X/10/8/1148/s1, Characteristics of synthesized compounds, Figure S1: Anti-HIV-1 activity of 3-carboxyacylbetulin phosphate and BVM in the tested concentration range, Figure S2: Cytotoxicity of betulin phosphate 2–5 in the tested concentration range, Table S1: Selected physicochemical properties of the compounds 2–5, Table S2: Scoring functions of the tested compounds (SARS-Cov-2 proteins), Figure S3: The lowest-energy docking poses of SARS-Cov-2 Mpro protein complexes with BVM (A) and betulinic acid (B), Figure S4: Visualization of interaction between BVM (A) and compound 4 (B) with SARS-Cov-2 RdRp, Figure S5: Docking pose of SARS-Cov-2 E protein complexes with BVM (A) and compound 6 (B), Figure S6: Visualization of interaction between betulinic acid (A) and BVM (B) with SARS-Cov-2 spike protein monomer, Figure S7: RMSD for atoms of protein (Mpro, RdRp, E) backbones (left) and ligands (right), Figure S8: RMSD for atoms of S protein backbones (left) and ligands (right), Figure S9: RMSD for atoms of E protein backbones without terminal residues, Table S3: Interactions of tested compounds with SARS-CoV-2 proteins (References [59,60] are cited in the supplementary materials).
