# Supplementary material for: Phosphate Derivatives of 3-Carboxyacylbetulin: SynThesis, In Vitro Anti-HIV and Molecular Docking Study
Source: Biomolecules. 2020 Aug 5;10(8):1148. doi: 10.3390/biom10081148 (PMC7464173; doi:10.3390/biom10081148)
Supplement: Supplementary file 1 [file biomolecules-10-01148-s001.pdf]

# Phosphate Derivatives of 3-Carboxyacylbetulin: Synthesis, In Vitro Anti-HIV and Molecular Docking Study

*Krzysztof Marciniec[1], Elwira Chrobak[1*], Aleksandra Dąbrowska[2], Ewa Bębenek[1], Monika Kadela-Tomanek[1], Paweł Pęcak[1], Stanisław Boryczka[1]*

[1] Department of Organic Chemistry, Faculty of Pharmaceutical Sciences in Sosnowiec, Medical University of Silesia in Katowice, 4 Jagiellońska Str., 41-200 Sosnowiec, Poland;

[2] National Medicines Institute, 30/34 Chełmska Str., 00-725 Warszawa, Poland

*Corresponding author

E-mail address: echrobak@sum.edu.pl

**Characteristics of synthesized compounds**

28-Diethoxyphosphorylbetulin **2** ($^{1}$H, $^{13}$C and $^{31}$P NMR spectra) ………………………..…………… **2**

28-Diethoxyphosphoryl-3-*O*-(3',3'-dimethylsuccinyl)betulin **3** ($^{1}$H, $^{13}$C, HSQC, HMBC, $^{31}$P NMR and HR MS spectra)........................................................................................................................................ **3**

28-Diethoxyphosphoryl-3-*O*-(3',3'-dimethylglutaryl)betulin **4** ($^{1}$H, $^{13}$C, $^{31}$P NMR and HR MS spectra)........................................................................................................................................ **6**

28-Diethoxyphosphoryl-3-*O*-(4',4'-dimethylglutaryl)betulin **5** ($^{1}$H, $^{13}$C, $^{31}$P NMR and HR MS spectra)........................................................................................................................................ **9**

**Figure S1.** Anti-HIV-1 activity of 3-carboxyacylbetulin phosphate and BVM in the tested concentration range ……………………………………………………........................................................... **10**

**Figure S2.** Cytotoxicity of betulin phosphate **2-5** in the tested concentration range …………………….. **11**

**Table S1**. Selected physicochemical properties of the compounds **2-5**…………………………....….. **12**

**Molecular docking to selected SARS-Cov-2 proteins**

**Table S2**. Scoring functions of the tested compounds (SARS-Cov-2 proteins)……………………….. **13**

**Figure S3.** The lowest-energy docking poses of SARS-Cov-2 M$^{pro}$ protein complexes with **BVM (A)** and betulinic acid (**B**)……………………………………………………………………………… **13**

**Figure S4**. Visualization of interaction between **BVM (A)** and compound **4** (**B**) with SARS-Cov-2 RdRp…………………………………………………...………………………………………… **14**

**Figure S5**. Docking pose of SARS-Cov-2 E protein complexes with **BVM (A)** and compound **6** (**B**)….. **15**

**Figure S6**. Visualization of interaction between betulinic acid **(A)** and **BVM** (**B**) with SARS-Cov-2 spike protein monomer……………………………………………………………………………….. **16**

**Figure S7.** RMSD for atoms of protein (Mpro, RdRp, E) backbones (left) and ligands (right)………..… **17**

**Figure S8.** RMSD for atoms of S protein backbones (left) and ligands (right)………………………..…. **18**

**Figure S9.** RMSD for atoms of E protein backbones without terminal residues……………………..….. **18**

**Table S3**. Interactions of tested compounds with SARS-CoV-2 proteins……………….…………….. **18**

**Characteristics of synthesized compounds**

***28-Diethoxyphosphorylbetulin 2***

$^1$H NMR

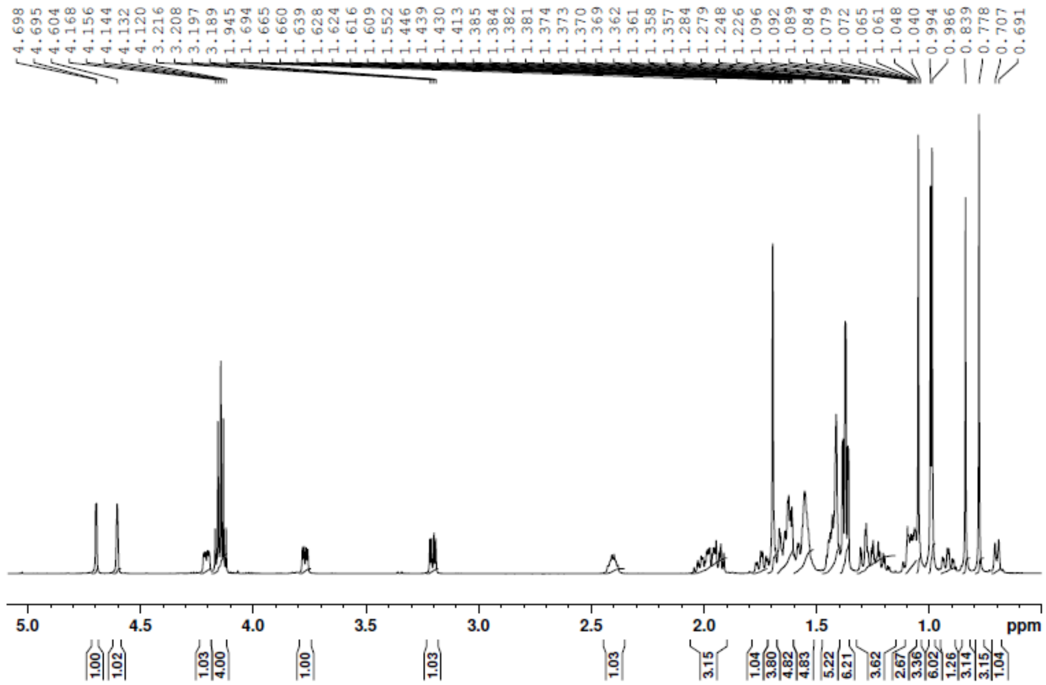

$^{13}$C NMR

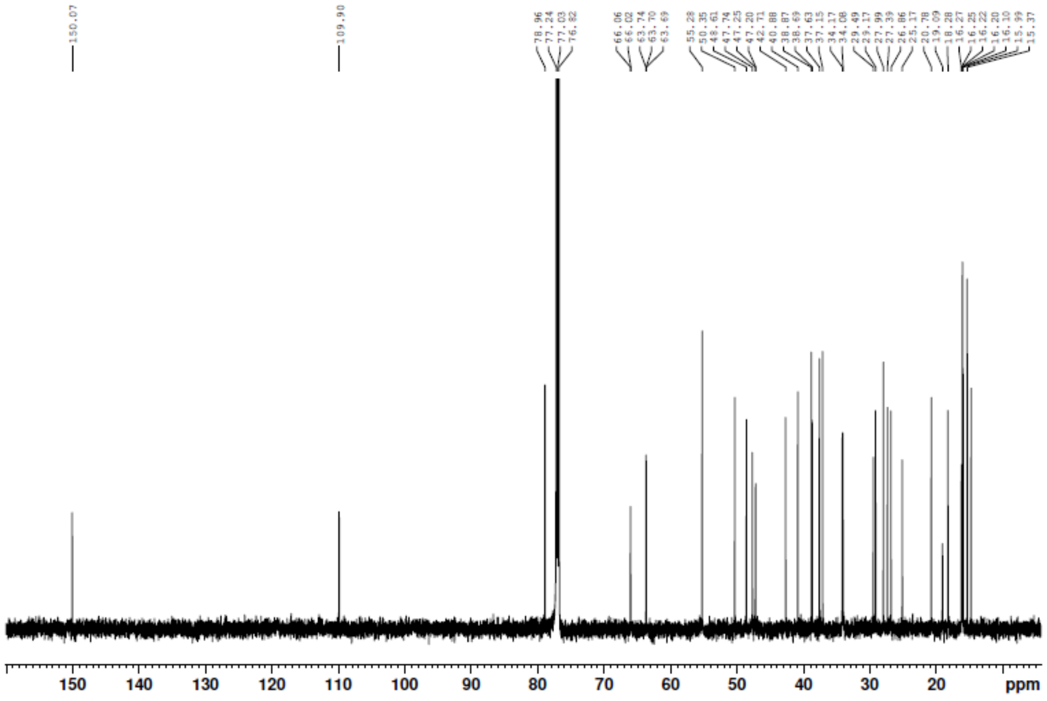

$^{31}$P NMR

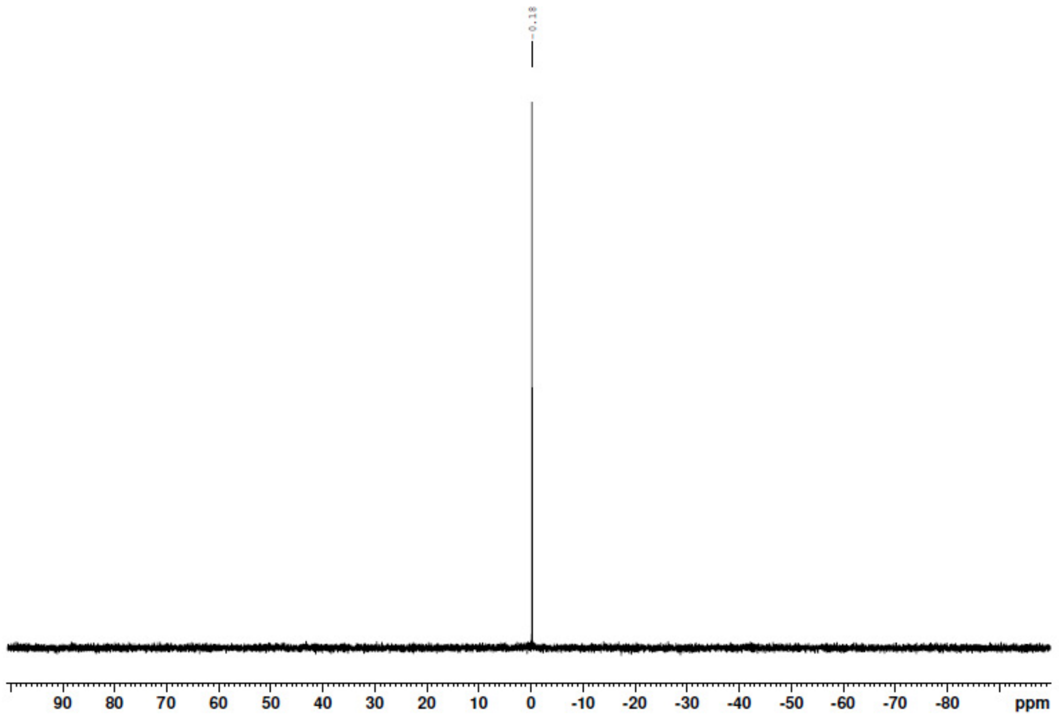


HR MS

## Mass Spectrum List Report

**Analysis Info**

| | | | |
|---|---|---|---|
| Analysis Name | D:\Data\PM_20171204\ECH 84.d | Acquisition Date | 6/26/2020 10:34:52 AM |
| Method | low_mass.m | Operator | KM |
| Sample Name | AN 40_18 | Instrument | impact II 1825265.10082 |
| Comment | | | |

**Acquisition Parameter**

| | | | | | |
|---|---|---|---|---|---|
| Source Type | APCI | Ion Polarity | Negative | Set Nebulizer | 2.0 Bar |
| Focus | Active | Set Capillary | 4000 V | Set Dry Heater | 200 °C |
| Scan Begin | 100 m/z | Set End Plate Offset | -500 V | Set Dry Gas | 5.0 l/min |
| Scan End | 2000 m/z | Set Charging Voltage | 2000 V | Set Divert Valve | Source |
| | | Set Corona | 2000 nA | Set APCI Heater | 450 °C |

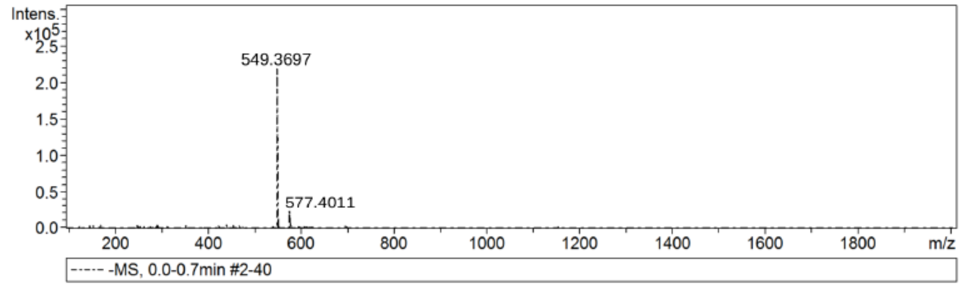


| # | m/z | Res. | S/N | I | I % | FWHM |
|---|---|---|---|---|---|---|
| 1 | 549.3697 | 41368 | 13537.2 | 214368 | 100.0 | 0.0118 |
| 2 | 577.4011 | 33399 | 1534.8 | 24697 | 11.5 | 0.0133 |

***28-Diethoxyphosphoryl-3-O-(3',3'-dimethylsuccinyl)betulin 3***

<sup></sup>$^{1}$H NMR

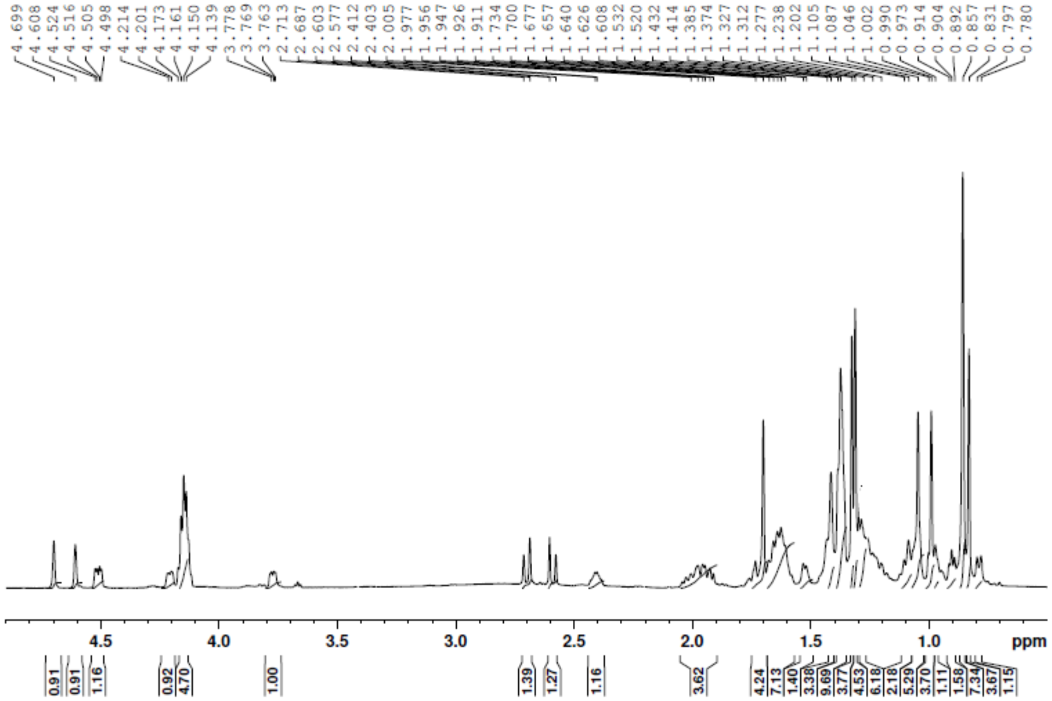

$^{13}$C NMR

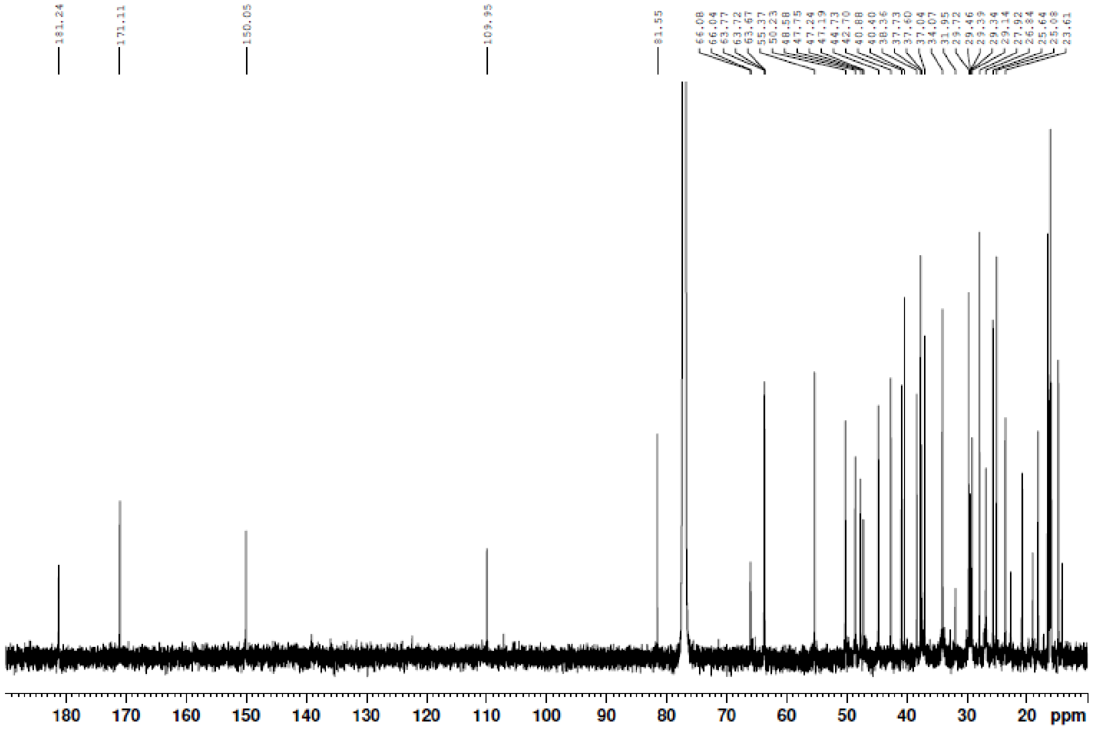

HSQC

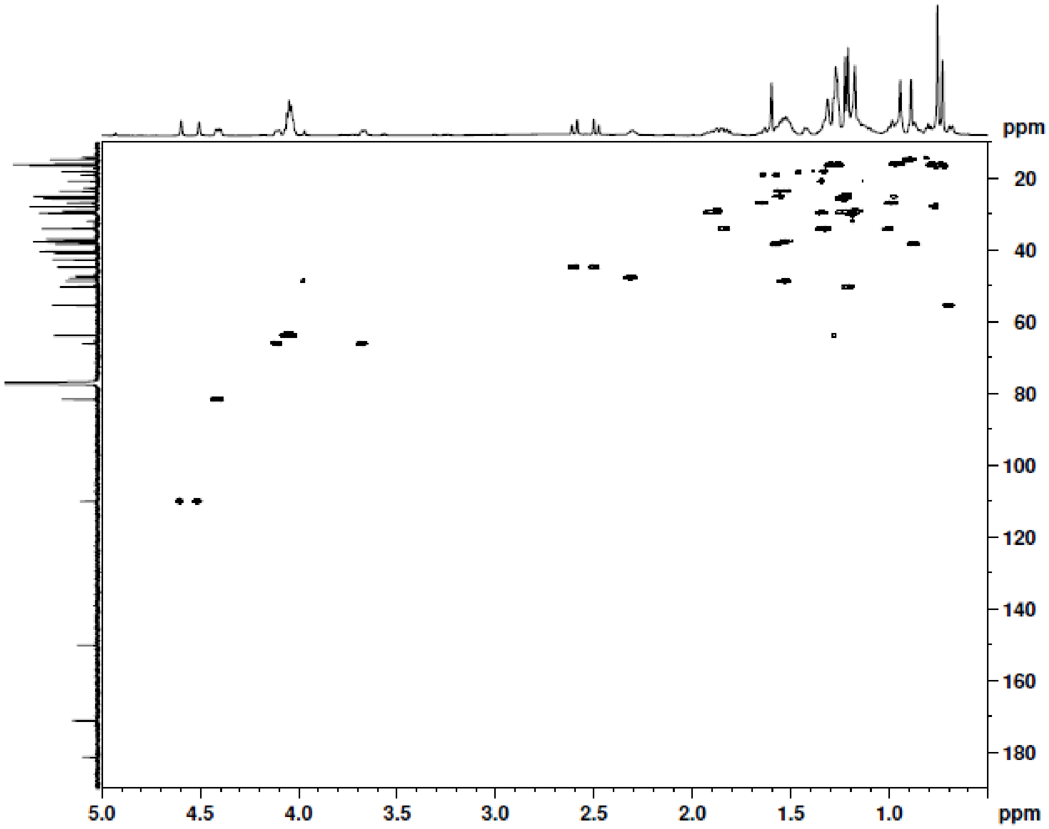


HMBC

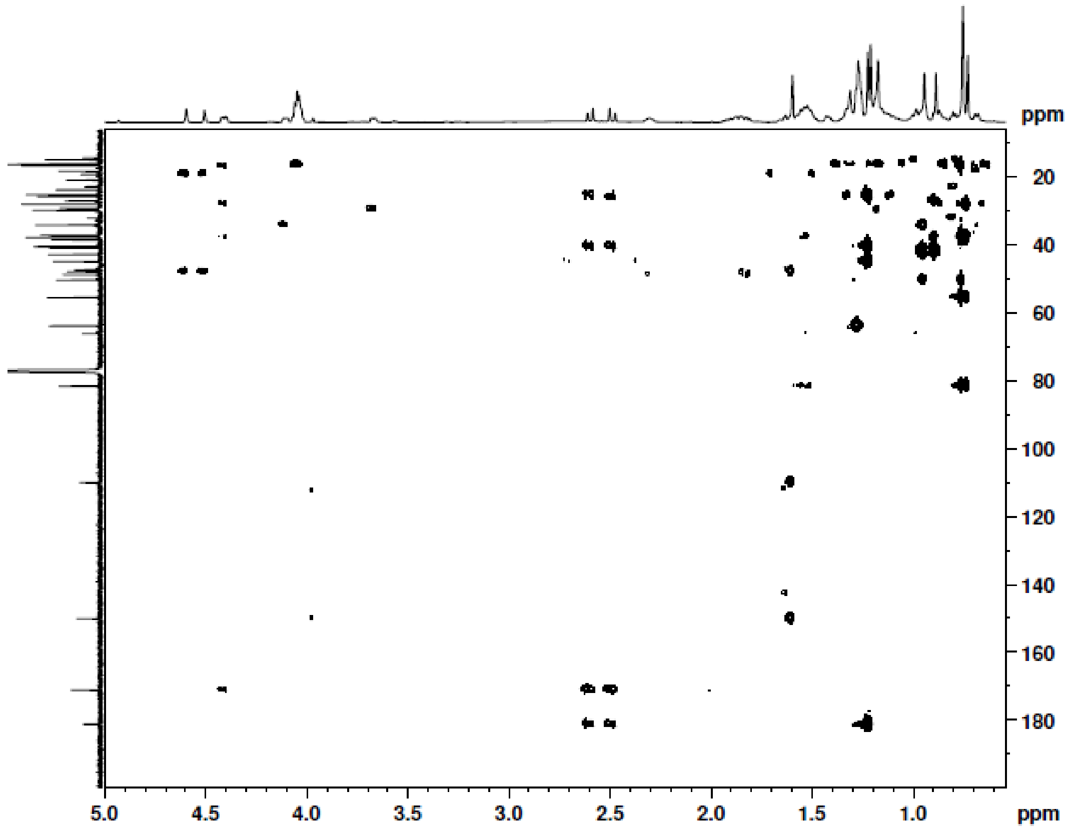
ppm
20
40
60
80
100
120
140
160
180
5.0
4.5
4.0
3.5
3.0
2.5
2.0
1.5
1.0
ppm

$^{31}$P NMR

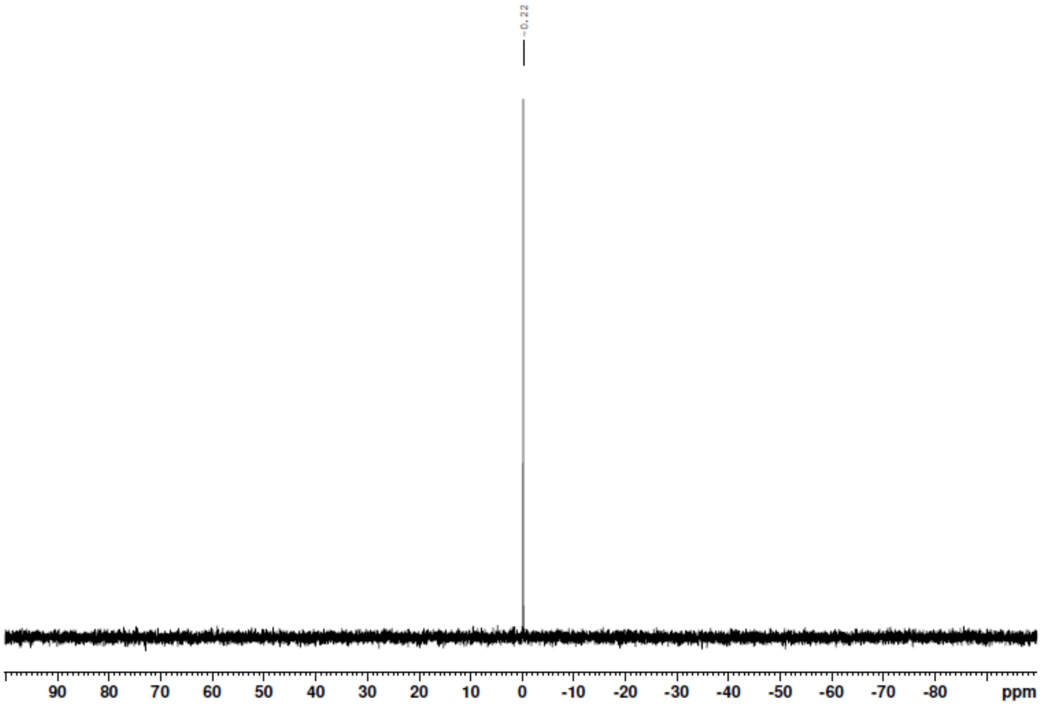
-0.22
90
80
70
60
50
40
30
20
10
0
-10
-20
-30
-40
-50
-60
-70
-80
ppm

HR MS

# Mass Spectrum List Report

**Analysis Info**

| | | | |
|---|---|---|---|
| Analysis Name | D:\Data\ECH245a.d | Acquisition Date | 6/26/2020 12:02:00 PM |
| Method | low_mass_20200619.m | Operator | KM |
| Sample Name | bzt | Instrument | impact II 1825265.10082 |
| Comment | | | |

**Acquisition Parameter**

| | | | | | |
|---|---|---|---|---|---|
| Source Type | APCI | Ion Polarity | Negative | Set Nebulizer | 2.0 Bar |
| Focus | Not active | Set Capillary | 4000 V | Set Dry Heater | 200 °C |
| Scan Begin | 100 m/z | Set End Plate Offset | -500 V | Set Dry Gas | 5.0 l/min |
| Scan End | 2500 m/z | Set Charging Voltage | 2000 V | Set Divert Valve | Source |
| | | Set Corona | 2000 nA | Set APCI Heater | 450 °C |

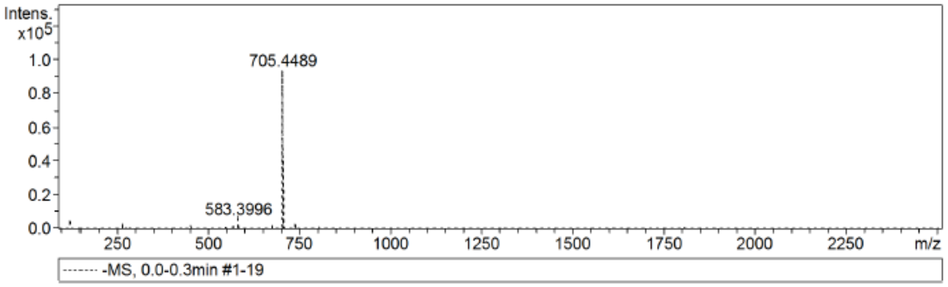


| # | m/z | Res. | S/N | I | I % | FWHM |
|---|---|---|---|---|---|---|
| 1 | 583.3996 | 16157 | 323.4 | 5768 | 6.2 | 0.0361 |
| 2 | 705.4489 | 16139 | 4693.5 | 93204 | 100.0 | 0.0437 |
| 3 | 741.4236 | 16463 | 182.2 | 3712 | 4.0 | 0.0450 |

***28-Diethoxyphosphoryl-3-O-(3',3'-dimethylglutaryl)betulin 4***

$^{1}$H NMR

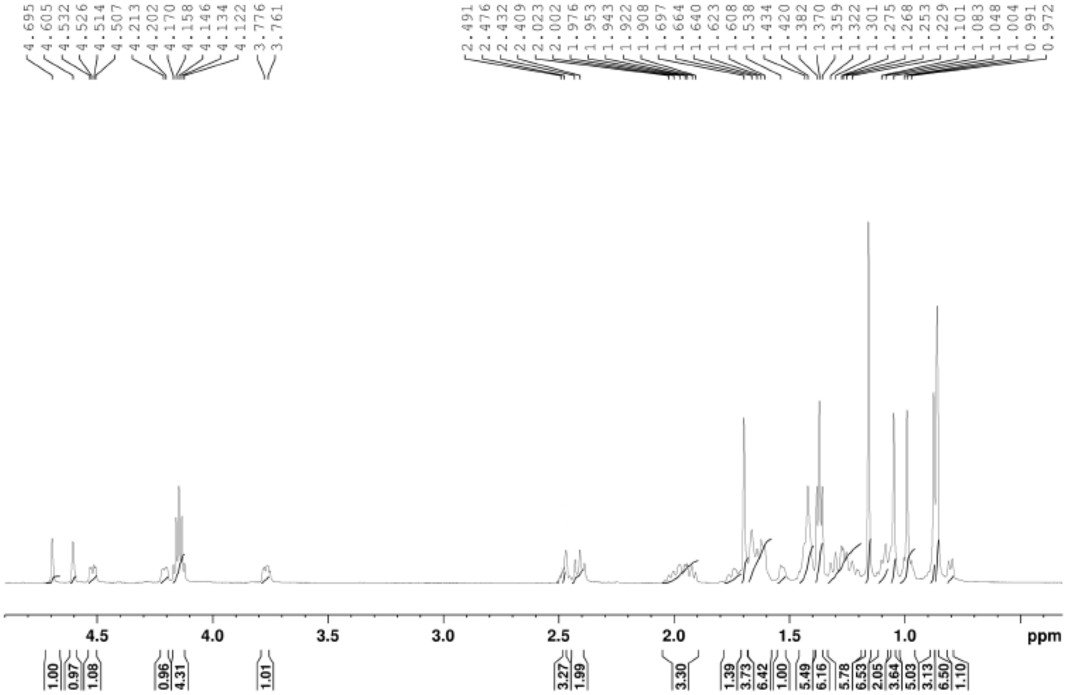

$^{13}$C NMR

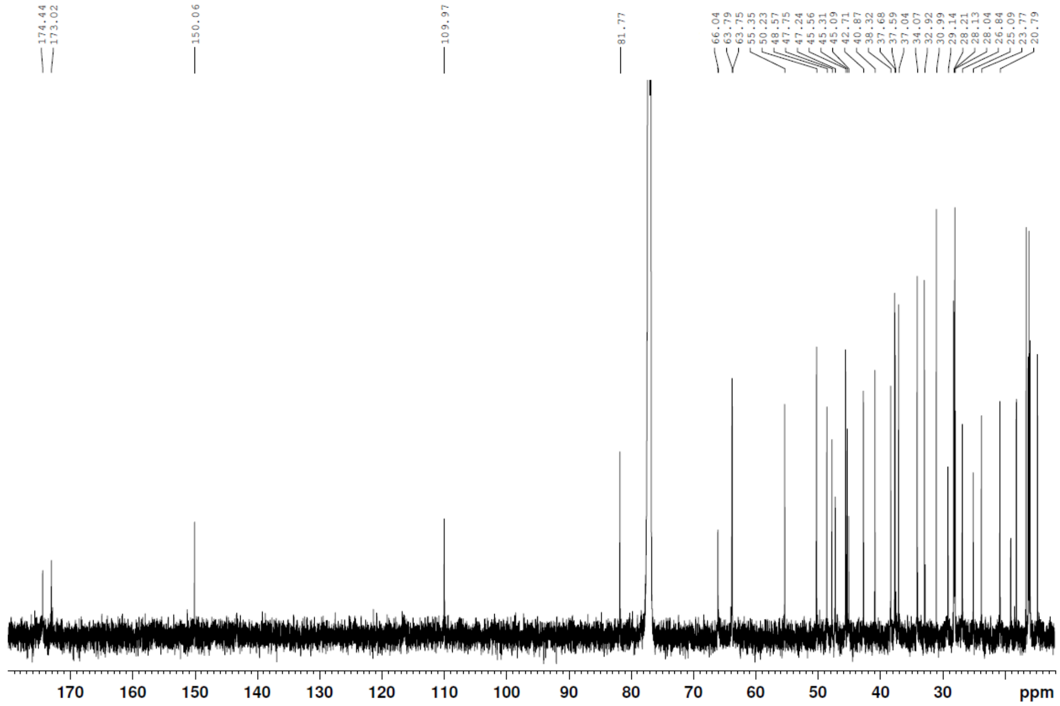

$^{31}P$ NMR

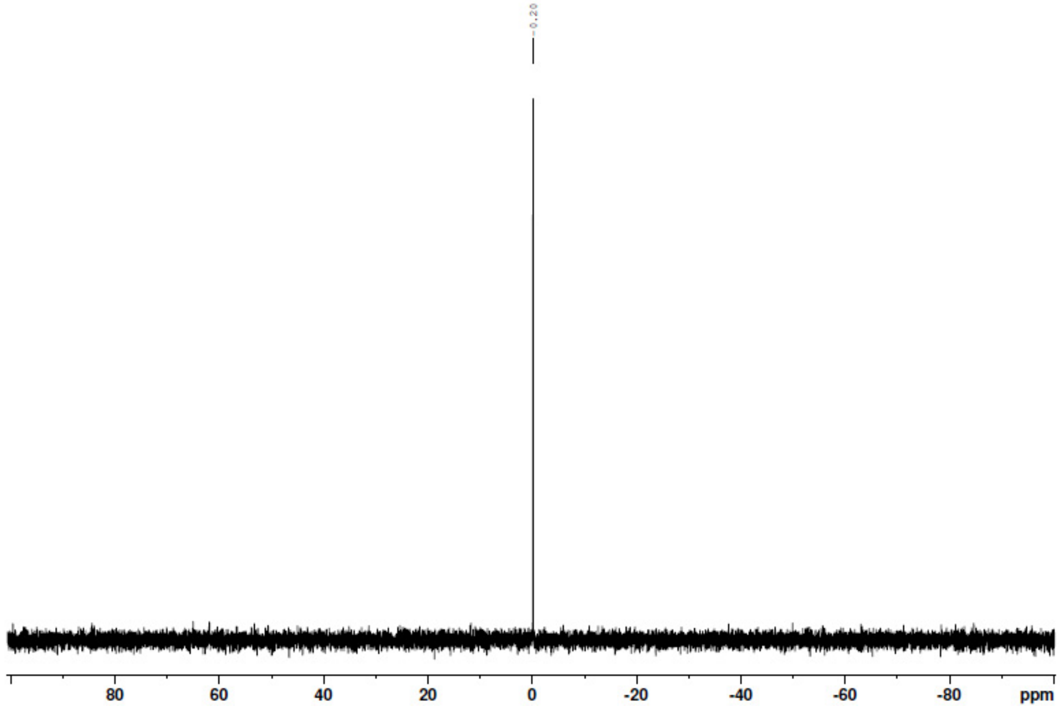

HR MS

## Mass Spectrum List Report

**Analysis Info**

| | | | |
|---|---|---|---|
| Analysis Name | D:\Data\ECH247.d | Acquisition Date | 6/26/2020 12:18:21 PM |
| Method | low_mass_20200619.m | Operator | KM |
| Sample Name | bzt | Instrument | impact II 1825265.10082 |
| Comment | | | |

**Acquisition Parameter**

| | | | | | |
|---|---|---|---|---|---|
| Source Type | APCI | Ion Polarity | Negative | Set Nebulizer | 2.0 Bar |
| Focus | Not active | Set Capillary | 4000 V | Set Dry Heater | 200 °C |
| Scan Begin | 100 m/z | Set End Plate Offset | -500 V | Set Dry Gas | 5.0 l/min |
| Scan End | 2500 m/z | Set Charging Voltage | 2000 V | Set Divert Valve | Source |
| | | Set Corona | 2000 nA | Set APCI Heater | 450 °C |

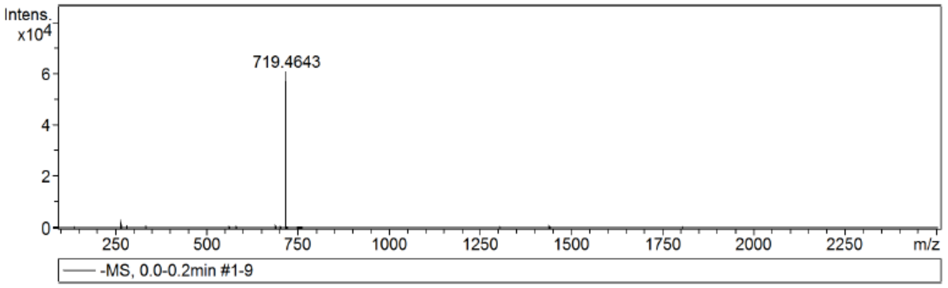


| # | m/z | Res. | S/N | I | I % | FWHM |
|---|---|---|---|---|---|---|
| 1 | 719.4643 | 16149 | 3536.3 | 60755 | 100.0 | 0.0446 |

### *28-Diethoxyphosphoryl-3-O-(4',4'-dimethylglutaryl)betulin 5*

$^{1}$H NMR

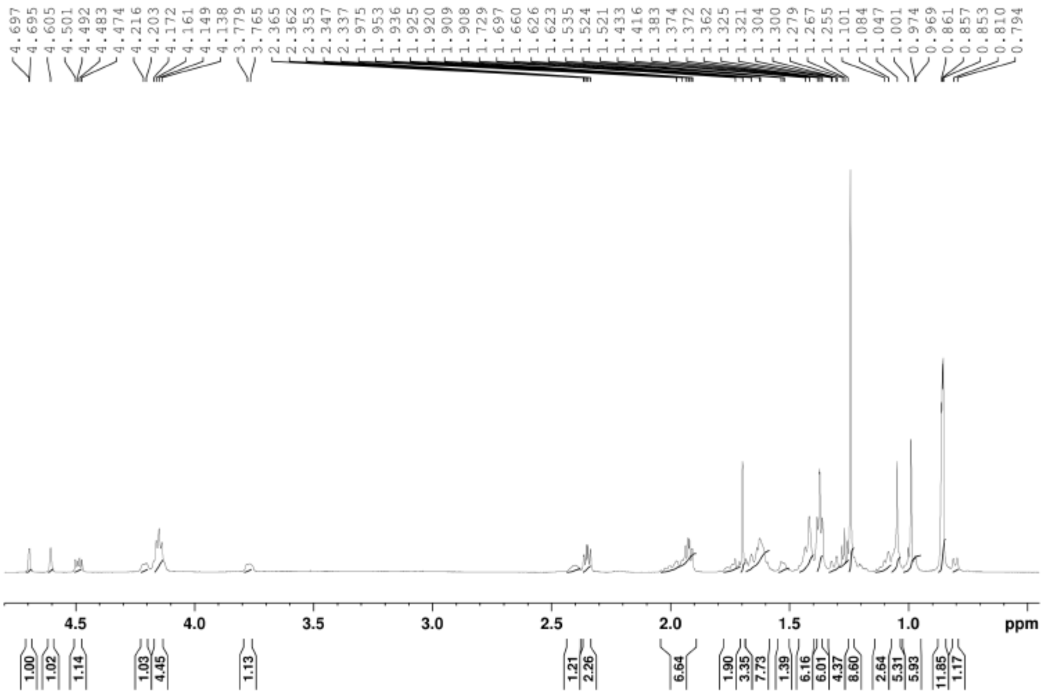


$^{13}$C NMR

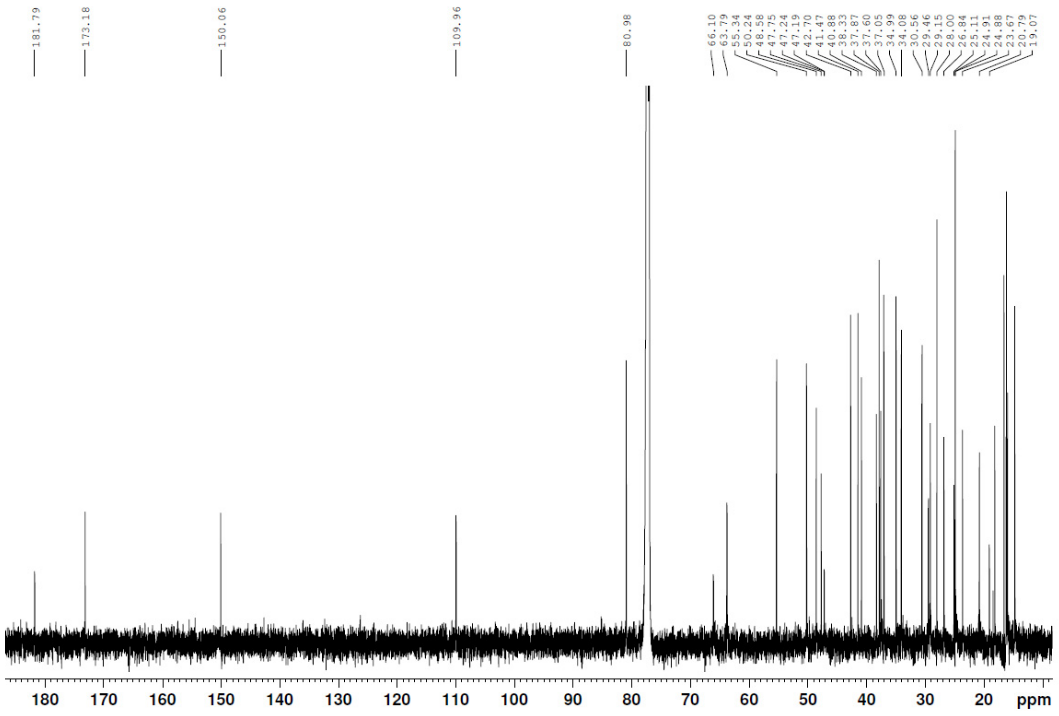
181.79
173.18
150.06
109.96
80.98
66.10
63.79
55.34
50.24
48.58
47.75
47.24
47.19
42.70
41.47
40.88
38.33
37.87
37.60
37.05
34.99
34.08
30.56
29.46
29.15
28.00
26.94
25.11
24.91
24.88
23.67
20.79
19.07
180 170 160 150 140 130 120 110 100 90 80 70 60 50 40 30 20 ppm

$^{31}$P NMR

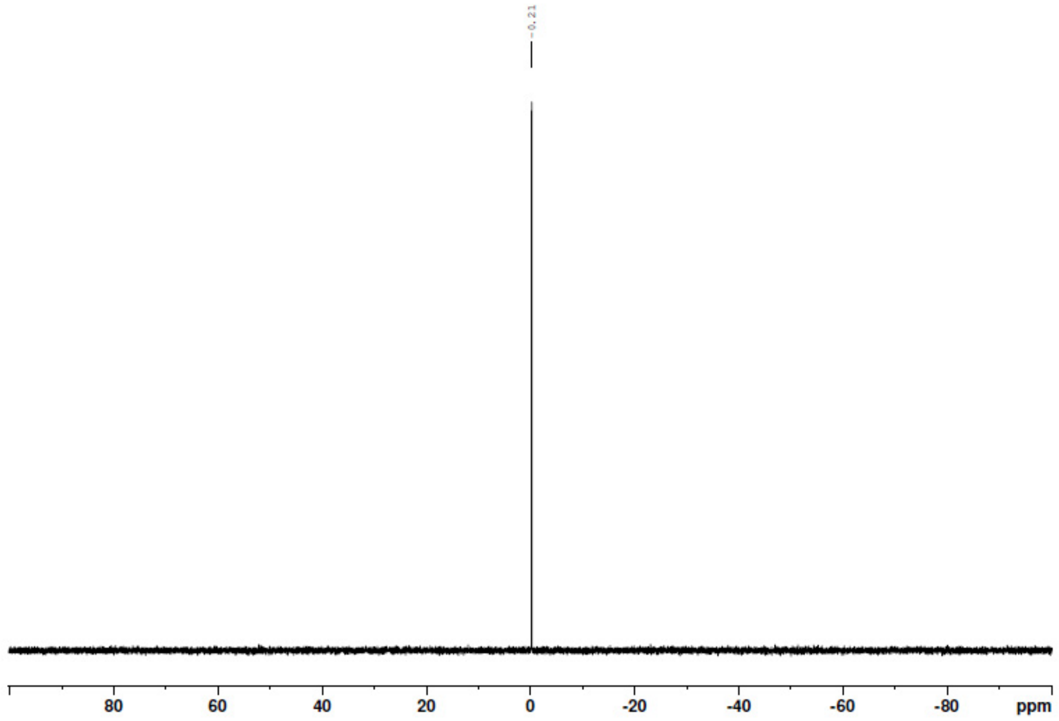
-0.21
80 60 40 20 0 -20 -40 -60 -80 ppm

HR MS

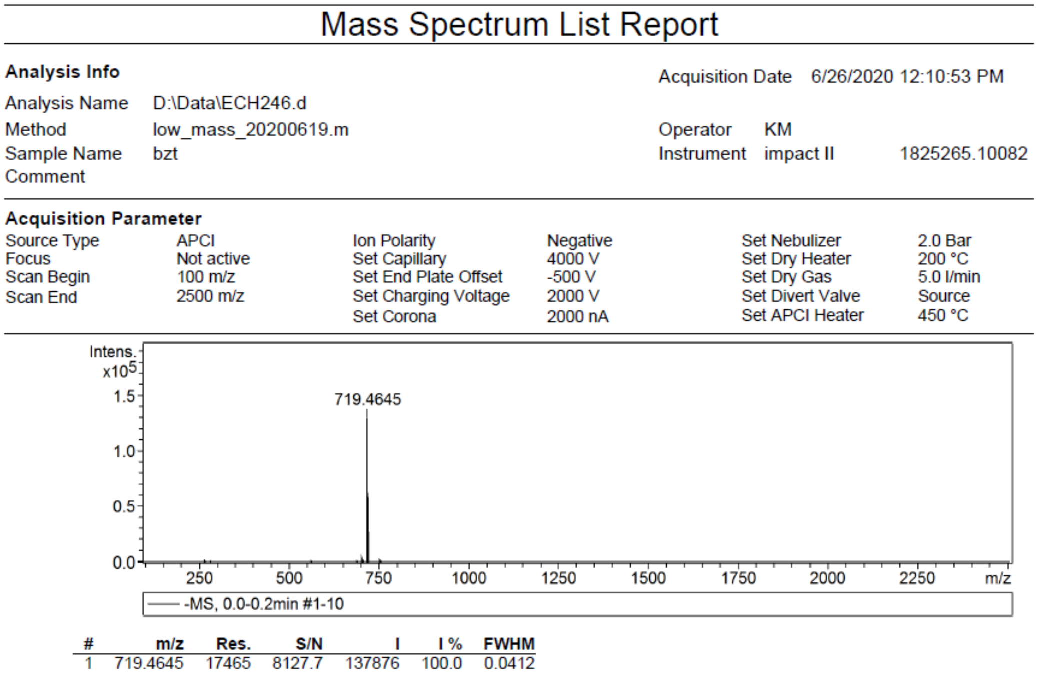

## Mass Spectrum List Report

**Analysis Info**

| | | | |
|---|---|---|---|
| Analysis Name | D:\Data\ECH246.d | Acquisition Date | 6/26/2020 12:10:53 PM |
| Method | low_mass_20200619.m | Operator | KM |
| Sample Name | bzt | Instrument | impact II 1825265.10082 |
| Comment | | | |

**Acquisition Parameter**

| | | | | | |
|---|---|---|---|---|---|
| Source Type | APCI | Ion Polarity | Negative | Set Nebulizer | 2.0 Bar |
| Focus | Not active | Set Capillary | 4000 V | Set Dry Heater | 200 °C |
| Scan Begin | 100 m/z | Set End Plate Offset | -500 V | Set Dry Gas | 5.0 l/min |
| Scan End | 2500 m/z | Set Charging Voltage | 2000 V | Set Divert Valve | Source |
| | | Set Corona | 2000 nA | Set APCI Heater | 450 °C |



| # | m/z | Res. | S/N | I | I % | FWHM |
|---|---|---|---|---|---|---|
| 1 | 719.4645 | 17465 | 8127.7 | 137876 | 100.0 | 0.0412 |

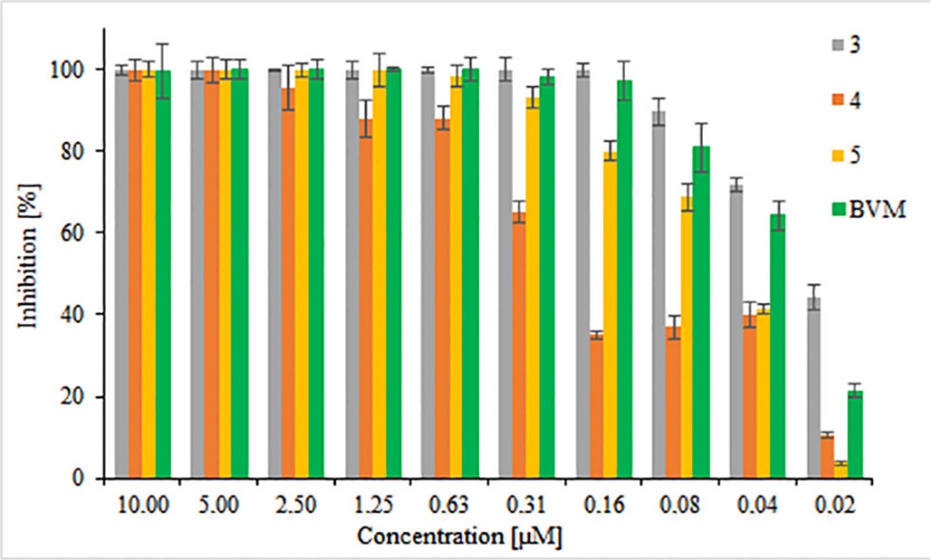


**Figure S1.** Anti-HIV-1 activity of 3-carboxyacylbetulin phosphate and **BVM** in the tested concentration range.

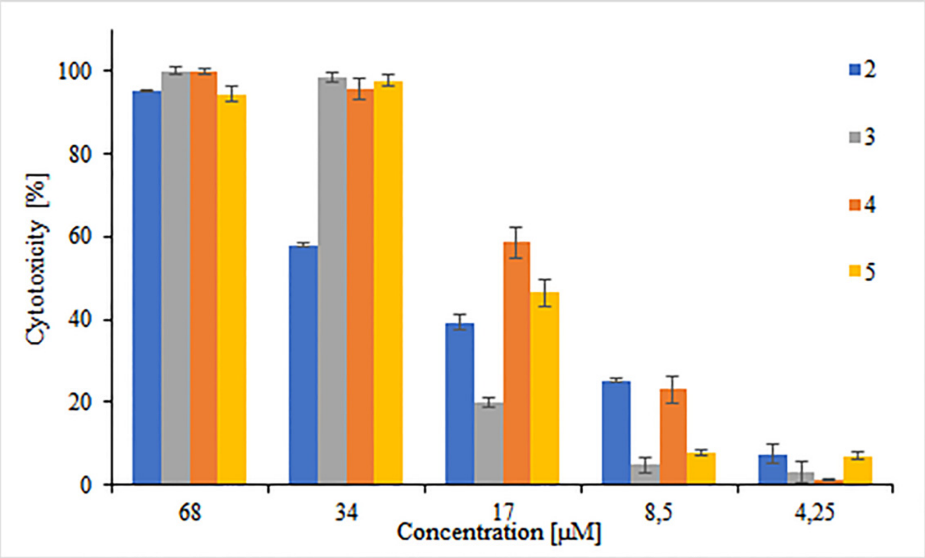


**Figure S2.** Cytotoxicity of betulin phosphate **2-5** in the tested concentration range.

**Table S1.** Selected physicochemical properties of the compounds **2-5**

| | Parametr | **2** | **3** | **4** | **5** |
|---|---|---|---|---|---|
| Biological parameters | Mol. Weight [g/mol] | 578.82 | 706.94 | 720.97 | 720.97 |
| | Log P | 9.20 | 10.25 | 10.65 | 10.65 |
| | nHBA | 1 | 7 | 7 | 7 |
| | nHBD | 5 | 1 | 1 | 1 |
| | nROTB | 8 | 12 | 13 | 13 |
| | TPSA | 65.00 | 108.38 | 108.38 | 108.38 |
| Absorption | Water solubility [log mol/L] | -6.225 | -4.507 | -4.275 | -4.275 |
| | Caco2 permeability [log Papp in $10^{-6}$ cm/s] | 0.879 | 0.536 | 0.52 | 0.52 |
| | Skin Permeability [log Kp] | -2.648 | -2.735 | -2.735 | -2.735 |
| Distribution | VDss (human) [log L/kg] | -0.654 | -1.274 | -1.324 | -1.324 |
| | BBB permeability [log BB] | -0.71 | -1.277 | -1.321 | -1.321 |
| | CNS permeability [log PS] | -1.247 | -2.017 | -2.011 | -2.011 |

Abbreviations: VDss - volume of distribution at steady state; BBB - blood–brain barrier permeability; CNS - central nervous system permeability.

## Molecular docking to selected SARS-Cov-2 proteins

**Table S2**. Scoring functions of the tested compounds (SARS-Cov-2 proteins).

| **Compound** | **SARS-Cov-2** ΔG [kcal/mol] | | | |
|---|---|---|---|---|
| | **M^pro^** | **RdRp** | **E protein** | **S protein** |
| **1** | -7.3 | -7.3 | -8.4 | -7.6 |
| **3** | -7.0 | -7.3 | -8.6 | -6.5 |
| **4** | -7.4 | -7.7 | -8.4 | -6.5 |
| **5** | -7.1 | -7.6 | -8.5 | -6.8 |
| **6** | -6.8 | -7.2 | -9.0 | -6.6 |
| **BVM** | -7.6 | -7.8 | -9.2 | -7.7 |
| betulinic acid | -7.6 | -7.4 | -8.9 | -8.0 |
| remdesivir | -7.1 | -7.0 | -8.1 | -6.8 |
| **HWH** | -6.1 | - | - | - |

**HWH** – **(***N*-[2-(5-fluoro-1*H*-indol-3-yl)ethyl]ethanamide**)** the native ligand for $M^{pro}$ (PDB ID:5R7Z)

The top docked pose of **BVM** in the binding site of $M^{pro}$ showed hydrophobic interactions with Cys145, Met49, and Pro168 (Table S3, Figure S3).

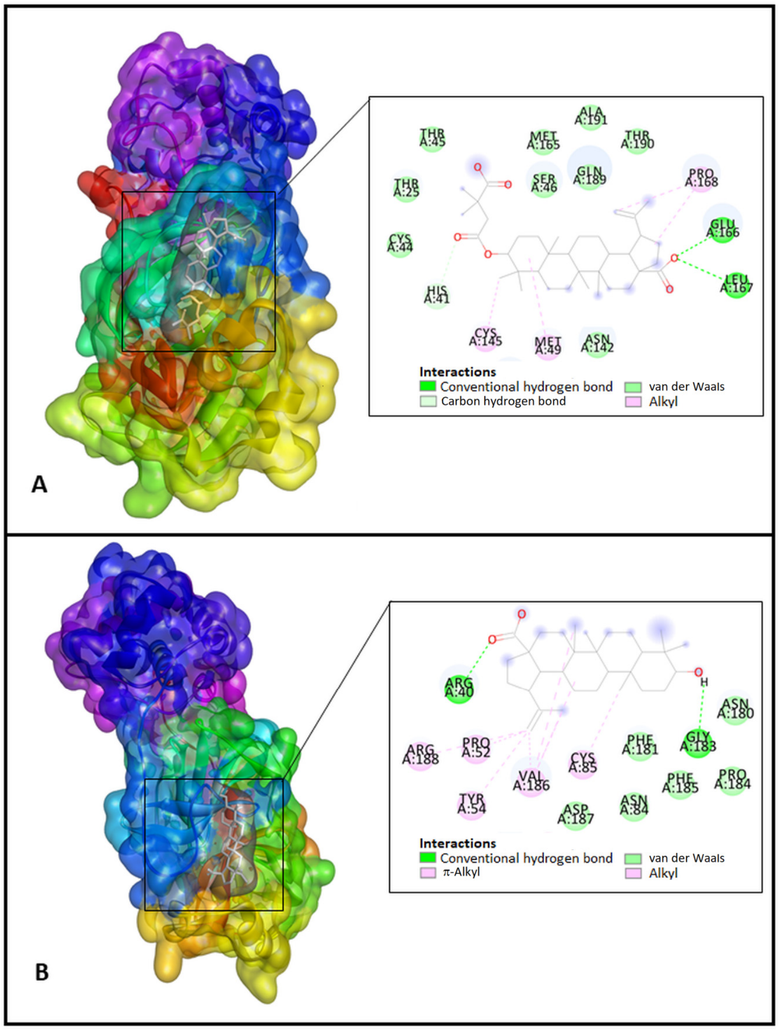


**Figure S3.** The lowest-energy docking poses of SARS-Cov-2 $M^{pro}$ protein complexes with **BVM (A)** and betulinic acid (**B**).

The carboxylate group of **BVM** in positions C28 were involved in hydrogen bonding with Glu166 and Leu167; the carboxylate group of betulinic acid were involved in hydrogen bonding with Arg40; and the hydroxyl group at C3 of betulinic acid was involved in hydrogen bonding with Gly183. Ligand **3** differs from **BVM** in having a diethoxyphosphoryl group at C28. The optimal docked pose of compound **3** was in a different place than for **BVM**, due to the three hydrogen bonds formed by the phosphoryl group (with Arg40, Tyr54, and Glu55) and four hydrogen bonds formed by the carboxylate group (with Arg105, Gly108, and Phe134). Like compound **3**, compounds **4** and **5** also formed a large number of conventional hydrogen bonds (Table S3). The phosphoryl and carboxylate groups of compound **4** were involved in hydrogen bonding with Arg40, Tyr54, Glu55, Arg105, and Gly183. Compound **5** formed, with the active site of $M^{pro}$, a network of hydrophobic interactions with Pro168, Met49, Pro168, Cys145, Pro168, and His41 residues in the hinge region area. For compound **5**, two hydrogen bonds with Ser46 and Gly170 residues are observed.

During docking of betulin, betulinic acid, and the phosphate ligands to RdRp, the optimally docked ligands were **BVM** and compound **4** (Table S2, Figure S4).

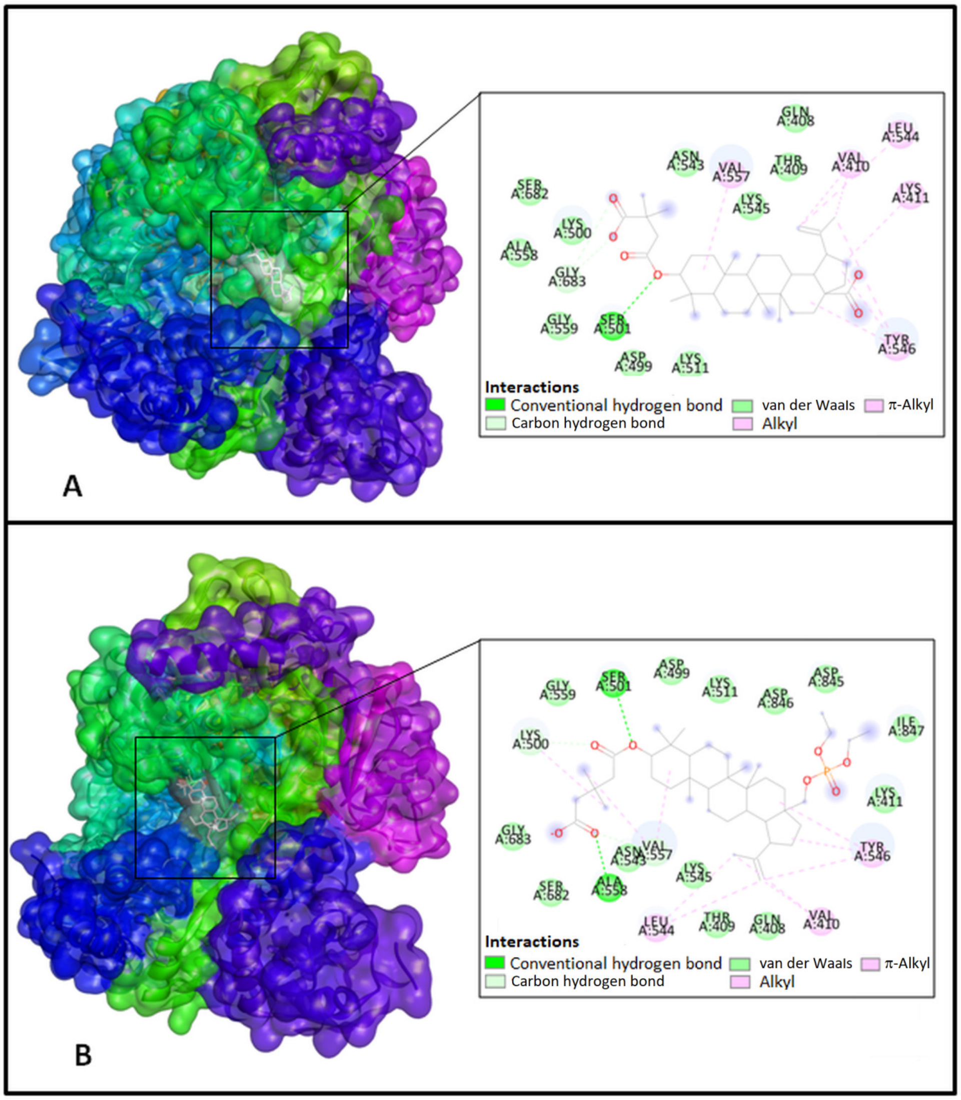


**Figure S4**. Visualization of interaction between **BVM (A)** and compound **4** (**B**) with SARS-Cov-2 RdRp.

All tested compounds showed a higher degree of fit compared to remdesivir, in the following order: **BVM** > **4** > **5** > betulinic acid > **1** = **3** > **6** (Table S2). All phosphate compounds (**3**, **4**, **5**, and **6**) and

**BVM** formed hydrogen bonds with Ser501, and derivatives **4** and **5** additionally formed hydrogen bonds with Ala558 residue. Moreover, all phosphate compounds interact with residues of Val557, Tyr546, and Val410, forming a hydrophobic site in the binding pocket (Table S3). According to the results from the molecular docking studies for RdRp, all triterpene derivatives (**1**, **3**, **4**, **5**, **6**, **BVM**, and betulinic acid) showed significantly better scores when compared with remdesivir (Table S2).

The active site pocket present in the SARS-CoV2 E protein was obtained from previously published literature [59]. The results of Gupta et al. revealed that 44 amino acids are involved in the formation of the active site, that is: Glu8, Thr11, Leu12, Val14, Asn15, Val17, Leu18, Leu19, Phe20, Leu21, Ala22, Phe23, Val24, Val25, Phe26, Leu27, Leu28, Val29, Thr30, Leu31, Ala32, Ile33, Leu34, Thr35, Ala36, Leu37, Arg38, Leu39, Ala40, Tyr42, Cys43, Ala44, Ile46, Val47, Val49, Leu51, Pro54, Val56, Tyr57, Ser60, Arg61, Lys63, Asn64, and Leu65 (Figure S5). Subsequently, a grid center was assigned to -0.496, 0.0, and 0.0 Å. It is pertinent to note that two amino acids, namely Val25 and Phe26, play a key role when interacting with ligands [59].

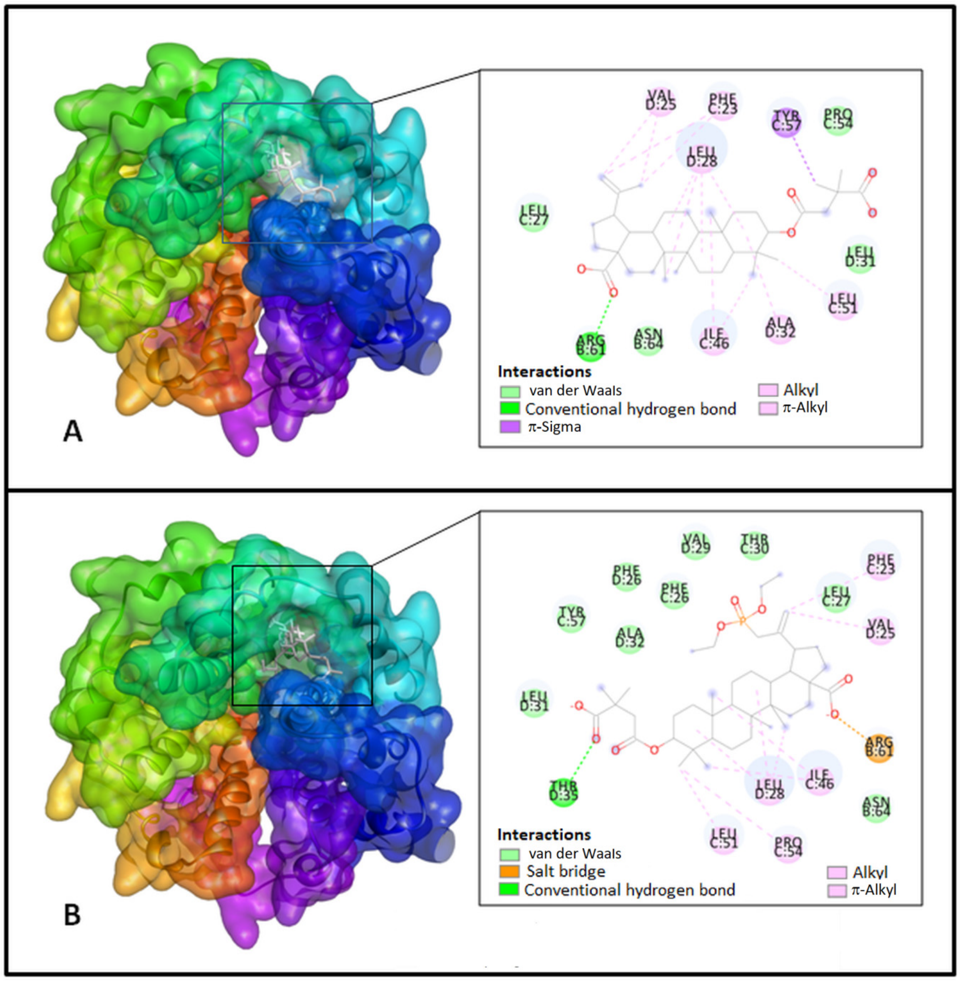


**Figure S5**. Docking pose of SARS-Cov-2 E protein complexes with **BVM (A)** and compound **6** (**B**).

According to the results of docking for E protein, all tested compounds showed a higher degree of fit compared to remdesivir, in the following order: **BVM** > **6** > betulinic acid > **3** > **5** > **1** = **4** (Table S2). The optimal docking poses of **BVM** and compound **6** inside the active side of E protein are presented in Figure S5. Betulin and its derivatives formed, with the active site of E protein, a network of hydrophobic interactions with amino acid residues in the hinge region area. For compounds **3**, **5**, and **6**, **BVM**, and betulinic acid only a single hydrogen bond is observed, but it can reach deep into the hydrophobic pocket region to form interactions with Phe20, Phe23, Val24, Val25, Phe26, Leu27, Val29, Leu31, Ala32, Leu34, Thr35, Ala40, Cys43, Ile46,Leu51, Pro54, Tyr57, Arg61, Asn64, and Leu65

amino acid residues (Table S3). As can be seen, the top docked poses of betulin and phosphate derivatives exhibit hydrophobic interactions with the Val25 and/or Phe26 amino acid residues. According to the literature, interactions with these amino acids play an important role in the regulation of envelope protein activity.

The entry of SARS-CoV into the host cell is mediated through the receptor binding domain (RBD) of the S1 subunit of the spike protein. This S1 subunit consists with two A and B domains. Human angiotensin-converting enzyme 2 (hACE2) receptor is binding target for the S1B domain. Residues 331 to 524 of the S glycoprotein have been recently identified as the RBD of the spike [60]. These findings makes RBD as he most important target for finding inhibitors, antibodies, or vaccines to stop entry of the virus into the host cell [56].

The analysis of the docking results presented in Table S2 shows that all phosphorated compounds exhibit higher (except compound **5**) binding energy values compared to remdesivir. The tested compounds demonstrate a degree of fit in the following order: betulinic acid > **BVM** > **1** > **5** = remdesivir > **6** > **3** = **4** (Table S2). The ligands optimally docked to spike protein were betulinic acid and BVM (Table S6). Figure S6 presents the possible interaction of betulinic acid inside the binding pocket of SARS-Cov-2 S protein after 2D analysis conducted in the Discovery Studio Visualizer.

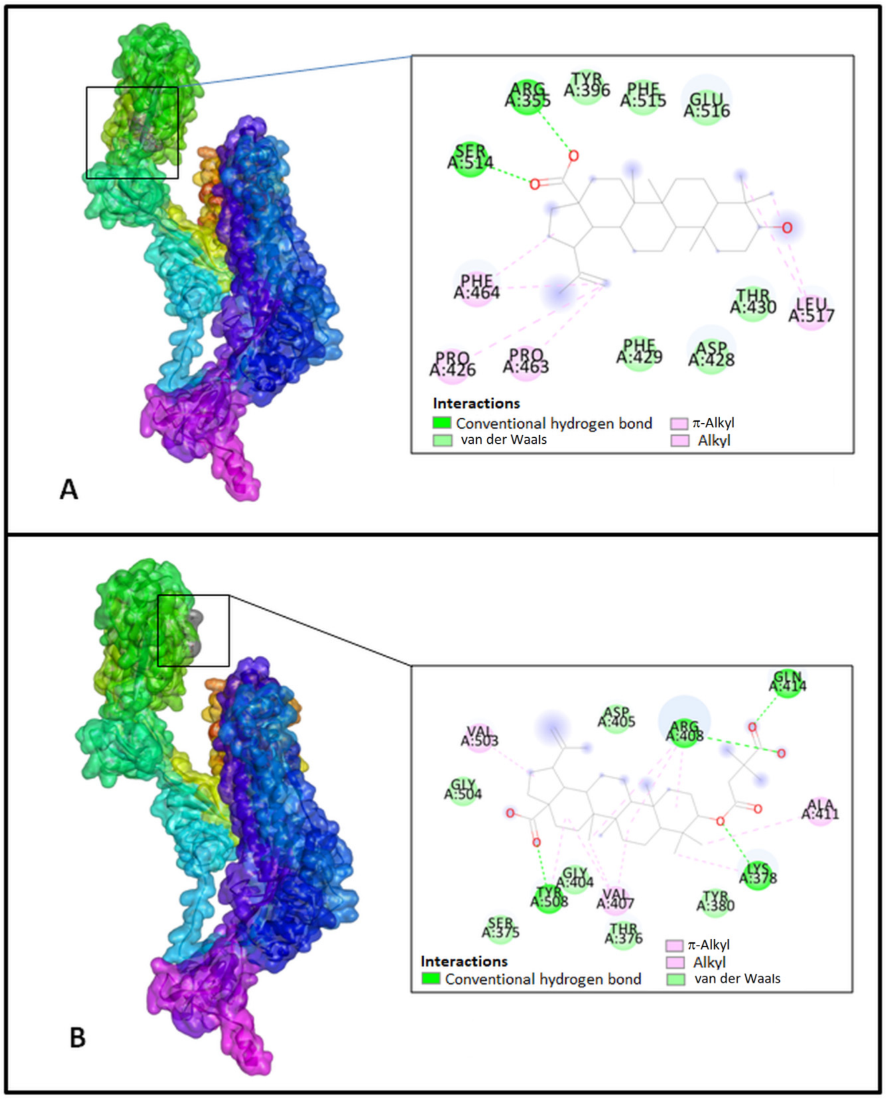


**Figure S6**. Visualization of interaction between betulinic acid **(A)** and **BVM** (**B**) with SARS-Cov-2 spike protein monomer.

Corresponding amino acids that are significantly involved in the hydrophobic interactions are as follows: Leu517, Pro426, Pro463 (alkyl), Phe464 (π-alkyl), and six amino acids (Van der Waals). Strong hydrogen bond interaction between Arg355 and Ser514 and the carboxylate group increase the stability of the ligand–receptor complex. In addition, with the active site of S protein, **BVM** formed a network of hydrogen bonds with Lys378, Arg408, Gln414, and Tyr508 residues. Amino acids that are significantly involved in the hydrophobic interactions are as follows: Val407, Arg408, Ala411, Val503, Lys378, Arg408 (alkyl), and Tyr508 (π-alkyl). The docking results indicate that betulin and phosphate derivatives **4** and **5** formed hydrogen bonds with Arg355, and that compounds **3** and **6** formed hydrogen bonds with Lys462 and Lys424, respectively.

As with CA-CTD-SP1, a MD simulation for SARS-CoV-2 protein-ligand complexes has been performed. Low fluctuations of RMSD of all proteins indicates, that they reached stable conformation. It seems that relatively big S protein needs longer simulation time, but at the end of studied simulation it stabilizes the RMSD. Visual detailed inspection of trajectories revealed, that hydrophilic terminal α-helices (C-terminal residues: Glu8, Thr9, Gly10, Thr11, Leu12, Ile13, Val14; N-terminal residues: Lys63, Asn64, Leu65) of E protein were considerably labile. This observation explains relatively high RMSD value of protein backbone (Fig. S7, S8).

$M^{pro}$

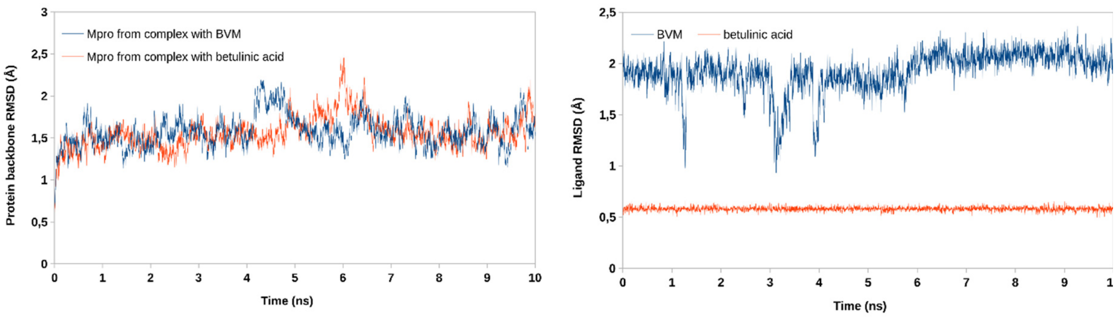


RdRp

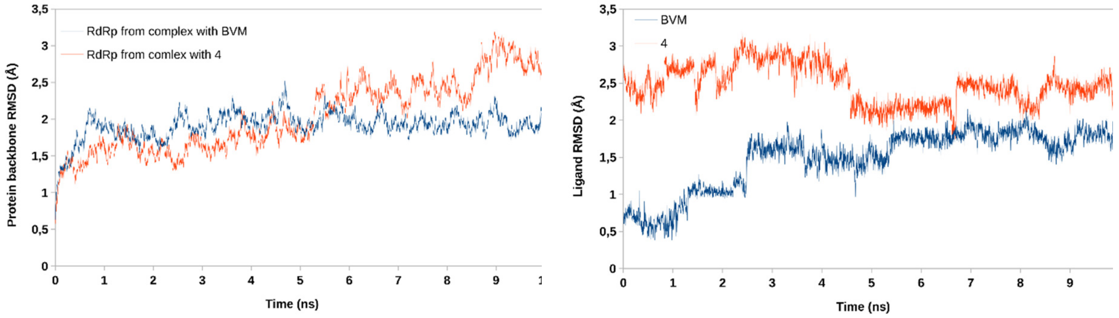


E protein

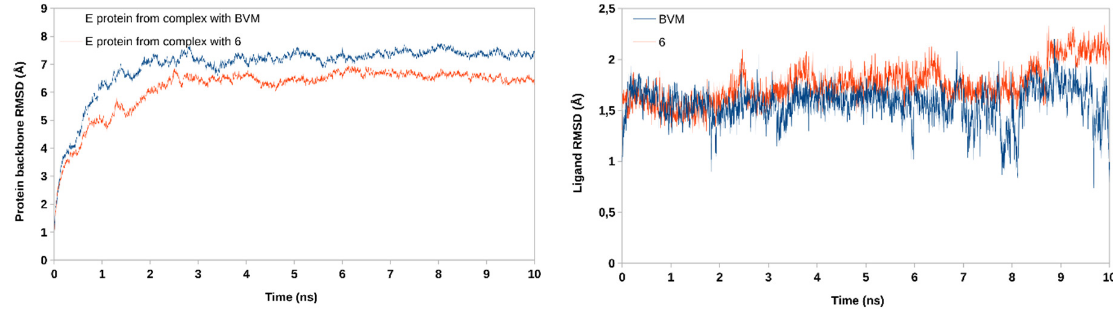


**Figure S7.** RMSD for atoms of protein ($M^{pro}$, RdRp, E) backbones (left) and ligands (right).

S protein

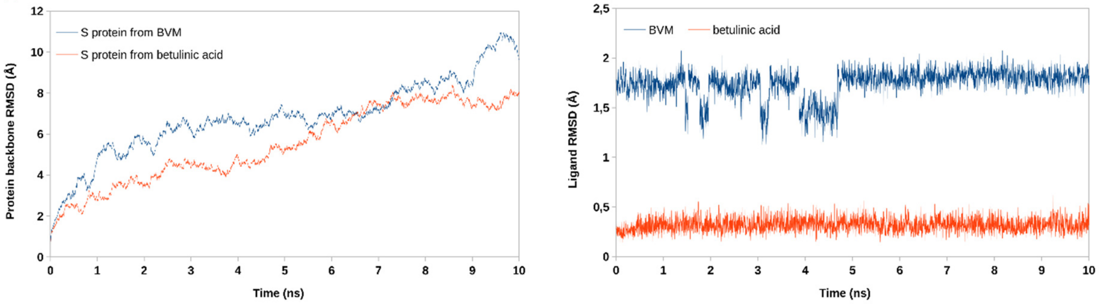


**Figure S8.** RMSD for atoms of S protein backbones (left) and ligands (right).

To verify this hypothesis we recalculated the RMSD of this protein backbone without residues listed above. This resulted in reducing backbone RMSD and thus prove our assumptions (Fig. S9).

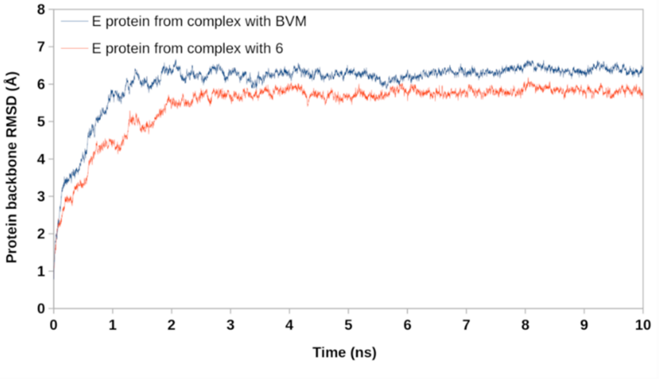


**Figure S9.** RMSD for atoms of E protein backbones without terminal residues.

All ligands showed RMSD below 2,5 Å, which demonstrate stability of the complexes. Further analysis of the results revealed, that betulinic acid demonstrated perfect binding stability in M$^{pro}$ and S protein. Unlike the other molecules studied, betulinic acid has only few small labile groups that could significantly affect RMSD. As result, RMSD values are lower and fluctuations are smaller than in other compounds.

**Table S3** Interactions of tested compounds with SARS-CoV-2 proteins

| **Protein** | | **Ligand** | | **Interaction** | |
|---|---|---|---|---|---|
| **Name** | **Residue** | **Name** | **Residue** | **Type** | **Distance [Å]** |
| M$^{pro}$ | Asn142 | **1** | C3 | carbon hydrogen bond | 3.24 |
| | Met49 | | C26 | alkyl-alkyl | 3.91 |
| | His41 | | C18 | π-alkyl | 5.28 |
| | Arg105 | **3** | carboxylate | attractive charge | 3.32 |
| | Arg40 | | phosphate | conventional hydrogen bond | 2.17 |
| | Tyr54 | | phosphate | conventional hydrogen bond | 2.64 |
| | Glu55 | | phosphate | conventional hydrogen bond | 2.06 |
| | Arg105 | | carboxylate | conventional hydrogen bond | 2.79 |
| | Gly183 | | carboxylate | conventional hydrogen bond | 2.36 |
| | Phe134 | | carboxylate | π-anion | 3.86 |
| | Val186 | | C29 | alkyl-alkyl | 4.89 |
| | Cys85 | | C25 | alkyl-alkyl | 4.50 |
| | Cys85 | | C26 | alkyl-alkyl | 5.22 |
| | Pro52 | | C29 | alkyl-alkyl | 5.02 |
| | Val186 | | C30 | alkyl-alkyl | 3.96 |

| | | | | | |
|---|---|---|---|---|---|
| | Arg188 | | C29 | alkyl-alkyl | 3.99 |
| | Val186 | | C29 | alkyl-alkyl | 4.13 |
| | Arg188 | | C30 | alkyl-alkyl | 4.69 |
| | Arg105 | **4** | carboxylate | attractive charge | 5.06 |
| | Arg40 | | phosphate | conventional hydrogen bond | 2.42 |
| | Tyr54 | | phosphate | conventional hydrogen bond | 2.56 |
| | Glu55 | | phosphate | conventional hydrogen bond | 2.21 |
| | Arg105 | | carboxylate | conventional hydrogen bond | 2.17 |
| | Gly183 | | carboxylate | conventional hydrogen bond | 2.01 |
| | Phe134 | | carboxylate | π-anion | 3.79 |
| | Val186 | | C30 | alkyl-alkyl | 4.84 |
| | Cys85 | | C25 | alkyl-alkyl | 4.49 |
| | Cys85 | | C26 | alkyl-alkyl | 5.14 |
| | Pro52 | | C29 | alkyl-alkyl | 5.01 |
| | Val186 | | C12 | alkyl-alkyl | 3.99 |
| | Arg188 | | C29 | alkyl-alkyl | 3.99 |
| | Val186 | | C30 | alkyl-alkyl | 4.36 |
| | Ser46 | **5** | carboxylate | conventional hydrogen bond | 2.05 |
| | Gly170 | | phosphate | conventional hydrogen bond | 3.07 |
| | Pro168 | | phosphate | carbon hydrogen bond | 3.57 |
| | Met49 | | C2 | alkyl-alkyl | 4.96 |
| | Pro168 | | C23 | alkyl-alkyl | 4.11 |
| | Cys145 | | C23 | alkyl-alkyl | 4.31 |
| | Pro168 | | C29 | alkyl-alkyl | 4.46 |
| | His41 | | C23 | π-alkyl | 5.31 |
| | His41 | | C2 | π-alkyl | 4.97 |
| | Asn119 | **6** | carboxylate | conventional hydrogen bond | 2.55 |
| | Asn142 | | carboxylate | conventional hydrogen bond | 2.84 |
| | Thr26 | | carboxylate | carbon hydrogen bond | 2.53 |
| | Cys145 | | C11 | alkyl-alkyl | 5.49 |
| | Met49 | | C25 | alkyl-alkyl | 4.78 |
| | Cys145 | | C26 | alkyl-alkyl | 4.82 |
| | His41 | | C12 | π-alkyl | 4.97 |
| | Glu166 | **BVM** | carboxylate | conventional hydrogen Bond | 3.34 |
| | Leu167 | | carboxylate | conventional hydrogen bond | 3.19 |
| | His41 | | ester | carbon hydrogen bond | 2.82 |
| | Met49 | | C4 | alkyl-alkyl | 5.19 |
| | Pro168 | | C21 | alkyl-alkyl | 4.23 |
| | Pro168 | | C29 | alkyl-alkyl | 4.56 |
| | Cys145 | | C24 | alkyl-alkyl | 4.53 |
| | Arg40 | **Betulinic acid** | carboxylate | conventional hydrogen bond | 2.21 |
| | Gly183 | | hydroxyl | conventional hydrogen Bond | 1.95 |
| | Val186 | | C29 | alkyl-alkyl | 4.83 |
| | Cys85 | | C25 | alkyl-alkyl | 4.57 |
| | Val186 | | C26 | alkyl-alkyl | 5.38 |
| | Pro52 | | C29 | alkyl-alkyl | 4.49 |
| | Val186 | | C12 | alkyl-alkyl | 4.61 |
| | Arg188 | | C29 | alkyl-alkyl | 4.06 |
| | Tyr54 | | C29 | π-alkyl | 5.30 |
| | Cys145 | **Remdesivir** | hydroxyl | conventional hydrogen bond | 2.49 |
| | Phe140 | | amine group | conventional hydrogen bond | 2.25 |
| | His164 | | hydroxyl | conventional hydrogen bond | 2.36 |
| | Gln189 | | phosphroamide | carbon hydrogen bond | 3.55 |
| | Thr190 | | ester group | carbon hydrogen bond | 3.35 |
| | Glu166 | | phenyl | π-cation | 4.51 |
| | Leu141 | | pirole ring | π-anion | 3.91 |
| | Leu141 | | 1,2,4-triazine ring | π-sigma | 3.63 |
| | His41 | **HWH** | fluorine | conventional hydrogen | 2.98 |
| | Glu166 | | amide | carbon hydrogen bond | 3.18 |
| | His164 | | fluorine | halogen (fluorine) | 3.64 |

| | | | | | |
|---|---|---|---|---|---|
| | Met49 | | benzene ring | π-sigma | 3.56 |
| | Met165 | | benzene ring | π-sulfur | 5.67 |
| | HIS41 | | benzene ring | π-π t-shaped | 4.94 |
| | MET49 | | pirole ring | π-alkyl | 5.01 |
| RdRp | Tyr455 | **1** | C23 | π-sigma | 3.85 |
| | Lys621 | | C26 | alkyl-alkyl | 5.39 |
| | Arg553 | | C24 | alkyl-alkyl | 4.18 |
| | Lys621 | | C23 | alkyl-alkyl | 4.70 |
| | Arg624 | | C23 | alkyl-alkyl | 4.93 |
| | Lys621 | | C7 | alkyl-alkyl | 3.89 |
| | Arg624 | | C25 | alkyl-alkyl | 4.74 |
| | Tyr455 | | C24 | π-alkyl | 4.15 |
| | Ser501 | **3** | ester group | conventional hydrogen bond | 2.42 |
| | Gly683 | | carboxylate | carbon hydrogen bond | 3.54 |
| | Val557 | | C1 | alkyl-alkyl | 5.18 |
| | Val410 | | C29 | alkyl-alkyl | 4.77 |
| | Leu544 | | C29 | alkyl-alkyl | 5.32 |
| | Val410 | | C30 | alkyl-alkyl | 3.71 |
| | Tyr546 | | C30 | π-alkyl | 5.22 |
| | Tyr546 | | C21 | π-alkyl | 4.69 |
| | Tyr546 | | C17 | π-alkyl | 5.32 |
| | Ser501 | **4** | ester group | conventional hydrogen bond | 2.40 |
| | Ala558 | | carboxylate | conventional hydrogen bond | 2.29 |
| | Val557 | | C'A | alkyl-alkyl | 5.09 |
| | Lys500 | | C'B | alkyl-alkyl | 3.82 |
| | Val557 | | C3 | alkyl-alkyl | 3.89 |
| | Val410 | | C29 | alkyl-alkyl | 3.72 |
| | Leu544 | | C30 | alkyl-alkyl | 5.49 |
| | Val410 | | C30 | alkyl-alkyl | 4.88 |
| | Leu544 | | C29 | alkyl-alkyl | 5.43 |
| | Tyr546 | | C29 | π-alkyl | 5.36 |
| | Tyr546 | | C21 | π-alkyl | 4.66 |
| | Tyr546 | | C17 | π-alkyl | 5.41 |
| | Ser501 | **5** | ester group | conventional hydrogen bond | 2.86 |
| | Ser501 | | ester group | conventional hydrogen bond | 2.68 |
| | Ala558 | | carboxylate | conventional hydrogen bond | 2.45 |
| | Lys411 | | C21 | alkyl-alkyl | 5.24 |
| | Val557 | | C1 | alkyl-alkyl | 5.02 |
| | Val410 | | C30 | alkyl-alkyl | 3.93 |
| | Tyr546 | | C30 | π-alkyl | 5.11 |
| | Tyr546 | | C21 | π-alkyl | 4.37 |
| | Tyr546 | | C17 | π-alkyl | 4.83 |
| | Ser501 | **6** | ester group | conventional hydrogen bond | 2.64 |
| | Lys411 | | phosphonate | carbon hydrogen bond | 3.78 |
| | Gly683 | | carboxylate | carbon hydrogen bond | 3.48 |
| | Val557 | | C5 | alkyl-alkyl | 5.18 |
| | Val410 | | C29 | alkyl-alkyl | 3.85 |
| | Tyr546 | | C29 | π-alkyl | 5.26 |
| | Tyr546 | | C21 | π-alkyl | 4.35 |
| | Tyr546 | | C17 | π-alkyl | 5.02 |
| | Ser501 | **BVM** | ester | conventional hydrogen bond | 2.58 |
| | Gly683 | | carboxylate | carbon hydrogen bond | 3.38 |
| | Gly683 | | carboxylate | carbon hydrogen bond | 3.62 |
| | Lys411 | | C21 | alkyl-alkyl | 5.29 |
| | Val557 | | C1 | alkyl-alkyl | 5.23 |
| | Val410 | | C29 | alkyl-alkyl | 4.57 |
| | Leu544 | | C29 | alkyl-alkyl | 5.01 |
| | Val410 | | C30 | alkyl-alkyl | 3.73 |
| | Tyr546 | | C30 | π-alkyl | 5.45 |
| | Tyr546 | | C21 | π-alkyl | 4.83 |

| | | | | | |
|---|---|---|---|---|---|
| | Tyr546 | | C16 | π-alkyl | 5.26 |
| | Arg553 | **Betulinic acid** | carboxylate | conventional hydrogen bond | 2.44 |
| | Lys551 | | carboxylate | carbon hydrogen bond | 3.54 |
| | Lys551 | | carboxylate | carbon hydrogen bond | 3.63 |
| | Lys621 | | C21 | alkyl-alkyl | 5.39 |
| | Lys500 | **Remdesivir** | cyanide group | conventional hydrogen bond | 1.98 |
| | Arg569 | | cyanide group | conventional hydrogen bond | 2.03 |
| | Tyr689 | | N4 of 1,2,4-triazine ring | conventional hydrogen bond | 2.13 |
| | Ser682 | | phosphoramide | conventional hydrogen bond | 2.38 |
| | Thr687 | | phenyl | π-sigma | 2.12 |
| | Ala685 | | pirole ring | alkyl-alkyl | 3.58 |
| | Lys500 | | buthyl | alkyl-alkyl | 3.69 |
| | Val557 | | buthyl | alkyl-alkyl | 4.24 |
| | Ala688 | | phenyl | π-alkyl | 4.44 |
| E protein | Leu27 | **1** | C30 | alkyl-alkyl | 4.83 |
| | Leu28 | | C26 | alkyl-alkyl | 4.84 |
| | Ala32 | | C23 | alkyl-alkyl | 4.29 |
| | Leu28 | | C25 | alkyl-alkyl | 5.25 |
| | Val29 | | C24 | alkyl-alkyl | 3.72 |
| | Leu28 | | C11 | alkyl-alkyl | 3.93 |
| | Leu27 | | C26 | alkyl-alkyl | 4.82 |
| | Leu65 | | C29 | alkyl-alkyl | 5.31 |
| | Val24 | | C30 | alkyl-alkyl | 4.53 |
| | Leu27 | | C10 | alkyl-alkyl | 4.91 |
| | Phe20 | | C21 | π-alkyl | 5.24 |
| | Phe23 | | C16 | π-alkyl | 5.31 |
| | Cys43 | **3** | ester | conventional hydrogen bond | 2.88 |
| | Leu27 | | C26 | alkyl-alkyl | 5.39 |
| | Val25 | | C30 | alkyl-alkyl | 5.35 |
| | Val25 | | C21 | alkyl-alkyl | 3.98 |
| | Leu28 | | C15 | alkyl-alkyl | 4.68 |
| | Val29 | | C30 | alkyl-alkyl | 4.89 |
| | Ile46 | | C23 | alkyl-alkyl | 3.97 |
| | Leu34 | | C23 | alkyl-alkyl | 3.80 |
| | Cys43 | | C23 | alkyl-alkyl | 5.29 |
| | Ile46 | | C24 | alkyl-alkyl | 4.46 |
| | Leu34 | | C25 | alkyl-alkyl | 3.98 |
| | Leu27 | | C12 | alkyl-alkyl | 4.52 |
| | Leu28 | | C27 | alkyl-alkyl | 4.72 |
| | Val29 | | C29 | alkyl-alkyl | 4.38 |
| | Val29 | | C12 | alkyl-alkyl | 4.72 |
| | Val25 | | C25 | π-alkyl | 4.51 |
| | Val29 | | C26 | π-alkyl | 4.54 |
| | Phe23 | | C21 | π-alkyl | 4.33 |
| | Leu34 | **4** | carboxylate | carbon hydrogen bond | 2.72 |
| | Leu27 | | C26 | alkyl-alkyl | 5.47 |
| | Val25 | | C29 | alkyl-alkyl | 3.94 |
| | Leu28 | | C27 | alkyl-alkyl | 4.69 |
| | Val29 | | C29 | alkyl-alkyl | 4.83 |
| | Ala32 | | C23 | alkyl-alkyl | 5.01 |
| | Ala32 | | C24 | alkyl-alkyl | 4.48 |
| | Ile46 | | C23 | alkyl-alkyl | 4.07 |
| | Leu34 | | C24 | alkyl-alkyl | 5.18 |
| | Cys43 | | C24 | alkyl-alkyl | 3.46 |
| | Ile46 | | C24 | alkyl-alkyl | 4.09 |
| | Leu34 | | C25 | alkyl-alkyl | 5.20 |
| | Leu27 | | C12 | alkyl-alkyl | 4.43 |
| | Leu28 | | C15 | alkyl-alkyl | 3.90 |
| | Val29 | | C27 | alkyl-alkyl | 4.66 |

| | | | | | |
|---|---|---|---|---|---|
| | Leu39 | | Cxx | alkyl-alkyl | 4.28 |
| | Val25 | | C21 | alkyl-alkyl | 4.36 |
| | Val29 | | C12 | alkyl-alkyl | 4.63 |
| | Val29 | | C30 | alkyl-alkyl | 4.93 |
| | Phe23 | | C21 | π-alkyl | 4.50 |
| | Phe26 | | C30 | π-alkyl | 4.47 |
| | Cys43 | **5** | ester | conventional hydrogen bond | 2.85 |
| | Val24 | | phosphate | carbon hydrogen bond | 2.89 |
| | Leu27 | | C26 | alkyl-alkyl | 5.38 |
| | Val25 | | C30 | alkyl-alkyl | 5.31 |
| | Val25 | | C21 | alkyl-alkyl | 3.99 |
| | Leu28 | | C17 | alkyl-alkyl | 4.81 |
| | Val29 Ile46 | | C29 | alkyl-alkyl | 4.88 |
| | Leu34 | | C23 | alkyl-alkyl | 3.85 |
| | Le46 | | C23 | alkyl-alkyl | 5.43 |
| | Leu34 | | C24 | alkyl-alkyl | 3.58 |
| | Leu27 | | C25 | alkyl-alkyl | 3.78 |
| | Leu28 | | C12 | alkyl-alkyl | 5.34 |
| | Val29 | | C16 | alkyl-alkyl | 4.33 |
| | Val29 | | C27 | alkyl-alkyl | 3.94 |
| | Val25 | | C12 | alkyl-alkyl | 4.60 |
| | Val29 | | C17 | alkyl-alkyl | 4.71 |
| | Phe23 | | C30 | alkyl-alkyl | 4.38 |
| | Phe26 | | C21 | alkyl-alkyl | 4.65 |
| | | | C29 | π-alkyl | 4.45 |
| | Arg61 | **6** | carboxylate | salt bridge;attractive charge | 2.18 |
| | Thr35 | | carboxylate | conventional hydrogen bond | 2.45 |
| | Leu28 | | C27 | alkyl-alkyl | 5.16 |
| | Leu28 | | C12 | alkyl-alkyl | 4.84 |
| | Ile46 | | C24 | alkyl-alkyl | 3.89 |
| | Leu51 | | C23 | alkyl-alkyl | 3.92 |
| | Pro54 | | C23 | alkyl-alkyl | 4.94 |
| | Ile46 | | C25 | alkyl-alkyl | 4.49 |
| | Leu28 | | C4 | alkyl-alkyl | 4.59 |
| | Arg61 | **BVM** | carboxylate | conventional hydrogen bond | 2.21 |
| | Ala32 | | ester | carbon hydrogen bond | 2.95 |
| | Tyr57 | | Cxx | pi-sigma | 3.86 |
| | Leu28 | | C12 | alkyl-alkyl | 4.91 |
| | Leu28 | | C27 | alkyl-alkyl | 5.47 |
| | Leu28 | | C11 | alkyl-alkyl | 4.92 |
| | Ala32 | | C2 | alkyl-alkyl | 5.22 |
| | Leu51 | | C23 | alkyl-alkyl | 4.40 |
| | Ile46 | | C24 | alkyl-alkyl | 4.05 |
| | Ile46 | | C25 | alkyl-alkyl | 4.72 |
| | Leu28 | | C1 | alkyl-alkyl | 4.42 |
| | Val25 | | C29 | alkyl-alkyl | 4.55 |
| | Val25 | | C30 | alkyl-alkyl | 4.21 |
| | Phe23 | | C29 | π-alkyl | 4.72 |
| | Phe23 | | C30 | π-alkyl | 4.54 |
| | Thr35 | **Betulinic acid** | carboxylate | conventional hydrogen bond | 2.58 |
| | Pro54 | | C27 | alkyl-alkyl | 4.65 |
| | Pro54 | | C15 | alkyl-alkyl | 5.31 |
| | Leu31 | | C26 | alkyl-alkyl | 4.98 |
| | Ala32 | | C25 | alkyl-alkyl | 4.26 |
| | Cys40 | | C29 | alkyl-alkyl | 3.92 |
| | Leu31 | | C15 | alkyl-alkyl | 5.16 |
| | Leu51 | | C24 | alkyl-alkyl | 4.69 |
| | Leu28 | | C23 | alkyl-alkyl | 4.49 |
| | Leu28 | | C24 | alkyl-alkyl | 4.81 |
| | Leu31 | | C6 | alkyl-alkyl | 3.61 |

| | | | | | |
|---|---|---|---|---|---|
| | Pro54 | | C6 | alkyl-alkyl | 4.87 |
| | Cys40 | | C21 | alkyl-alkyl | 4.28 |
| | Tyr57 | | C23 | π-alkyl | 4.92 |
| | Tyr57 | | C7 | π-alkyl | 4.19 |
| | Tyr57 | | C27 | π-alkyl | 5.35 |
| | Arg61 | | cyanide | conventional hydrogen bond | 2.16 |
| | Tyr57 | | ester | conventional hydrogen bond | 2.86 |
| | Leu28 | | phosphoramide | conventional hydrogen bond | 2.68 |
| | Asn64 | | hydroxyl | conventional hydrogen bond | 3.02 |
| | Val29 | | phenyl | pi-sigma | 2.58 |
| | Cys43 | | 1,2,4-trazine ring | pi-sulfur | 3.78 |
| | | | phenyl | | |
| | Phe23 | | ethyl | pi-pi t-shaped | 5.41 |
| | Leu31 | **Remdesivir** | ethyl | alkyl-alkyl | 5.22 |
| | Pro54 | | ethyl | alkyl-alkyl | 4.43 |
| | Tyr57 | | ethyl | π-alkyl | 4.40 |
| | Tyr57 | | phenyl | π-alkyl | 4.61 |
| | Leu27 | | pirole ring | π-alkyl | 3.97 |
| | Leu27 | | phenyl | π-alkyl | 4.88 |
| | Ala32 | | pirole ring | π-alkyl | 5.06 |
| | Ala32 | | 1,2,4-triazine ring | π-alkyl | 5.03 |
| | Ile 46 | | | π-alkyl | 5.17 |
| | Arg355 | | carboxylate | conventional hydrogen bond | 2.20 |
| | Ser514 | | carboxylate | conventional hydrogen bond | 2.12 |
| | Leu517 | | C23 | alkyl-alkyl | 5.36 |
| | Leu517 | | C24 | alkyl-alkyl | 4.77 |
| | Pro426 | **1** | C29 | alkyl-alkyl | 4.84 |
| | Pro463 | | C29 | alkyl-alkyl | 4.77 |
| | Phe464 | | C29 | π-alkyl | 4.86 |
| | Phe464 | | C21 | π-alkyl | 4.84 |
| | Lys462 | | ester | conventional hydrogen bond | 2.43 |
| | Glu516 | | phosphate | carbon hydrogen bond | 3.06 |
| | Pro426 | **3** | C27 | alkyl-alkyl | 5.24 |
| | Lys462 | | C23 | alkyl-alkyl | 4.16 |
| | Pro463 | | C23 | alkyl-alkyl | 3.52 |
| | Pro426 | | C7 | alkyl-alkyl | 4.09 |
| | Arg355 | | phosphate | conventional hydrogen bond | 2.73 |
| | Arg355 | | phosphate | conventional hydrogen bond | 2.49 |
| | Thr430 | | ester | conventional hydrogen bond | 2.51 |
| | Leu517 | | carboxylate | conventional hydrogen bond | 2.31 |
| | Leu518 | | carboxylate | conventional hydrogen bond | 2.84 |
| S protein | Glu516 | | carboxylate | carbon hydrogen bond | 3.06 |
| | Pro426 | **4** | C24 | alkyl-alkyl | 4.09 |
| | Leu517 | | Cxx | alkyl-alkyl | 4.59 |
| | Leu517 | | Cxx | alkyl-alkyl | 4.21 |
| | Tyr396 | | C15 | π-alkyl | 4.68 |
| | Phe464 | | C15 | π-alkyl | 5.32 |
| | Phe464 | | C6 | π-alkyl | 4.73 |
| | Arg355 | | phosphate | conventional hydrogen bond | 2.11 |
| | Thr430 | | carboxylate | conventional hydrogen bond | 2.57 |
| | Gly381 | | carboxylate | carbon hydrogen bond | 2.87 |
| | Pro426 | | C29 | alkyl-alkyl | 4.69 |
| | Pro463 | | C12 | alkyl-alkyl | 4.68 |
| | Leu518 | **5** | C23 | alkyl-alkyl | 4.33 |
| | Leu517 | | C23 | alkyl-alkyl | 5.20 |
| | Leu517 | | C24 | alkyl-alkyl | 4.19 |
| | Pro426 | | C29 | alkyl-alkyl | 4.26 |
| | Pro463 | | C29 | alkyl-alkyl | 3.85 |
| | Lys424 | **6** | carboxylate | attractive charge | 5.54 |
| | Asp428 | | phosphonate | attractive charge | 5.42 |

| | | | | | |
|---|---|---|---|---|---|
| | Asp427 | | carboxylate | conventional hydrogen bond | 2.07 |
| | Lys424 | | carboxylate | carbon hydrogen bond | 2.69 |
| | Pro426 | | carboxylate | carbon hydrogen bond | 2.49 |
| | Asp428 | | ethyl | carbon hydrogen bond | 3.54 |
| | Pro463 | | C27 | alkyl-alkyl | 4.30 |
| | Lys462 | | C23 | alkyl-alkyl | 4.34 |
| | Pro463 | | C2 | alkyl-alkyl | 3.83 |
| | Pro426 | | C27 | alkyl-alkyl | 4.13 |
| | Pro463 | | C17 | alkyl-alkyl | 3.80 |
| | Phe464 | | C27 | alkyl-alkyl | 4.48 |
| | Phe464 | | C21 | π-alkyl | 4.43 |
| | Lys378 | **BVM** | ester | conventional hydrogen bond | 2.41 |
| | Arg408 | | carboxylate | conventional hydrogen bond | 2.56 |
| | Gln414 | | carboxylate | conventional hydrogen bond | 2.07 |
| | Tyr508 | | carboxylate | conventional hydrogen bond | 2.19 |
| | Val407 | | C27 | alkyl-alkyl | 4.88 |
| | Val407 | | C7 | alkyl-alkyl | 4.90 |
| | Arg408 | | C1 | alkyl-alkyl | 4.63 |
| | Arg408 | | C27 | alkyl-alkyl | 5.20 |
| | Ala411 | | C23 | alkyl-alkyl | 4.37 |
| | Val503 | | C21 | alkyl-alkyl | 5.32 |
| | Lys378 | | C24 | alkyl-alkyl | 3.67 |
| | Val407 | | C15 | alkyl-alkyl | 4.13 |
| | Arg408 | | C15 | alkyl-alkyl | 4.37 |
| | Tyr508 | | C16 | π-alkyl | 5.10 |
| | Arg355 | **Betulinic acid** | carboxyl | conventional hydrogen bond | 2.20 |
| | Ser514 | | carboxyl | conventional hydrogen bond | 2.12 |
| | Leu517 | | C23 | alkyl-alkyl | 5.36 |
| | Leu517 | | C24 | alkyl-alkyl | 4.77 |
| | Pro426 | | C29 | alkyl-alkyl | 4.84 |
| | Pro463 | | C29 | alkyl-alkyl | 4.77 |
| | Phe464 | | C29 | π-alkyl | 4.86 |
| | Phe464 | | C21 | π-alkyl | 4.84 |
| | Thr430 | **Remdesivir** | hydoxyl | conventional hydrogen bond | 2.71 |
| | Leu517 | | cyanide | conventional hydrogen bond | 2.22 |
| | Phe515 | | hydroxyl | conventional hydrogen bond | 2.05 |
| | Asp428 | | phosphoramide | conventional hydrogen bond | 2.48 |
| | Glu516 | | cyanide | carbon hydrogen bond | 2.68 |
| | Asp428 | | phosphoramide | carbon hydrogen bond | 3.09 |
| | Glu516 | | pirole ring | π-anion | 4.66 |
| | Glu516 | | 1,2,4-triazine ring | π-anion | 3.84 |
| | Phe464 | | phenyl | π-π t-shaped | 4.81 |
| | Pro426 | | phenyl | π-alkyl | 5.32 |
